# FBXW7 regulates DISC1 stability via the ubiquitin-proteosome system

**DOI:** 10.1038/mp.2017.138

**Published:** 2017-07-20

**Authors:** K Yalla, C Elliott, J P Day, J Findlay, S Barratt, Z A Hughes, L Wilson, E Whiteley, M Popiolek, Y Li, J Dunlop, R Killick, D R Adams, N J Brandon, M D Houslay, B Hao, G S Baillie

**Affiliations:** 1 College of Veterinary Medical and Life Sciences, Institute of Cardiovascular and Medical Sciences, University of Glasgow, Glasgow, UK; 2 Institute of Psychiatry, Psychology and Neuroscience, King’s College, London, UK; 3 Neuroscience Research Unit, Pfizer Inc, Cambridge, MA, USA; 4 Department of Molecular Biology and Biophysics, University of Connecticut Health Centre, Farmington, CT, USA; 5 AstraZeneca, Neuroscience, Innovative Medicines & Early Development, Waltham, MA, USA; 6 Institute of Chemical Sciences, Heriot-Watt University, Edinburgh, UK; 7 Institute of Pharmaceutical Science, King’s College, London, UK

## Abstract

Disrupted in schizophrenia 1 (DISC1) is a multi-functional scaffolding protein that has been associated with neuropsychiatric disease. The role of DISC1 is to assemble protein complexes that promote neural development and signaling, hence tight control of the concentration of cellular DISC1 in neurons is vital to brain function. Using structural and biochemical techniques, we show for we believe the first time that not only is DISC1 turnover elicited by the ubiquitin proteasome system (UPS) but that it is orchestrated by the F-Box protein, FBXW7. We present the structure of FBXW7 bound to the DISC1 phosphodegron motif and exploit this information to prove that disruption of the FBXW7-DISC1 complex results in a stabilization of DISC1. This action can counteract DISC1 deficiencies observed in neural progenitor cells derived from induced pluripotent stem cells from schizophrenia patients with a DISC1 frameshift mutation. Thus manipulation of DISC1 levels via the UPS may provide a novel method to explore DISC1 function.

## Introduction

Disrupted in Schizophrenia 1 (DISC1) is a susceptibility gene for a range of psychiatric disorders and was identified in a Scottish family with a high frequency of mental illness.^1^ The many biological functions attributed to DISC1 include roles in neural development, neuronal differentiation/migration and synapse formation/maintenance (reviewed by Brandon and Sawa^2^). These all appear to occur by the ability of DISC1 to interact with a diverse set of signaling proteins located in distinct intracellular compartments, providing what has been termed the ‘DISC1 interactome’.^3^

Insights into the molecular mechanisms modulated by DISC1 only began to emerge following elucidation of the biology of DISC1-interacting proteins and protein complexes assembled by DISC1.^4^ As certain neuronal signaling complexes depend on DISC1 to assemble them with correct stoichiometry, it is clear that DISC1 levels must be maintained at the appropriate concentration in multiple cellular locations simultaneously. Little, however, is known about the processes that regulate the synthesis and degradation of DISC1.

Protein degradation by the proteosome and lysosome is a dynamic process that is crucial for neurodevelopment, synaptic plasticity and neuronal self-renewal.^5^ Indeed, the balance between protein synthesis and degradation in neurons is now regarded as a control point for regulation of processes that underpin learning and memory.^5^ Recent evidence suggests that the half-life of specific neuronal proteins can be profoundly influenced by synaptic activity, where the stoichiometry of such proteins is uniquely tailored in a manner that promotes memory formation. Crucial to this process is the compartmentalization of protein turnover at the synapse, which maintains optimal levels of the evolutionarily conserved macromolecular signaling complexes that are required for neural development and synaptic transmission.^6^ Interestingly, DISC1 protein levels are decreased in subjects carrying the *t*(1;11) DISC1 translocation,^7^ a DISC1 frameshift mutation^8, 9^ and in the lymphocytes of a small cohort of patients diagnosed with schizophrenia.^10^ DISC1 transcripts are also decreased in patients with bipolar disorder.^11^ In contrast, DISC1 levels appear unchanged in the hippocampus and dorsolateral prefrontal cortex of postmortem human brain samples extracted from patients with schizophrenia.^12^ In addition, one study has demonstrated that DISC1 transcript levels actually decrease in cells collected from schizophrenic patients treated for acute psychosis, suggesting that antipsychotics might potentially regulate the levels of DISC1 in the cell.^13^ These observations point to a key role for DISC1 in the development of a normally functioning brain. However, nothing is known about the molecular mechanisms that fine tune DISC1 levels in neurons.

Here, we demonstrate a novel mechanism for DISC1 turnover. This occurs through the ubiquitin-proteosome system (UPS) and involves a specific interaction between DISC1 and the E3 ubiquitin ligase, FBXW7, an F-box protein component of the cullin-RING ubiquitin E3 ligase superfamilies.^14^ The F-box proteins recruit specific substrates to the RING E3 core through their variable carboxy-terminal protein-interaction domains. Through complementary structural and biochemical approaches, we show that DISC1 contains a ‘phosphodegron’ (PD) binding site for FBXW7 and that a cell-permeable peptide mimic of this sequence can block UPS-mediated DISC1 degradation. Indeed, this peptide can restore DISC1 levels to normal levels in neural progenitor cells derived from induced pluripotent stem cells from schizophrenia/major depression patients with a DISC1 frameshift mutation (previously characterized by Wen *et al.*^9^).

## Materials and methods

### HEK293 cell culture

HEK293 cells were sourced from ATCC (CRL-1573) and cultured as described in the manufacturer’s protocol. DMEM (SIGMA) supplemented with 10% fetal bovine serum, 2 mM L-glutamine, 100 U μg^–1^ penicillin-streptomycin at 37 °C in a humidified atmosphere of 5% CO_2_. The cells were passaged at about 80% confluency. Cells were transfected with plasmid DNA using polyfect transfection reagent (Qiagen) according to the manufacturer’s instructions. HEK293 cells were treated with the pharmacological agents cycloheximide (C7698) and MG132 (M7449) (Sigma-Aldrich) or DISC1 disruptor peptides (custom synthesized by Severn Biotech) as described in the text.

### F-box siRNA library screen

ON Target PLUS siRNA libraries for F-box (G-105625-05) were purchased from Dharmacon, Thermofisher Scientific. HEK293 cells were plated at a density of 3 × 10^3^ cells per well in 6-well plates. Twenty-four hours after incubation, the cells were transfected with control non-specific siRNA or the test siRNA from the above library at a final concentration of 25 nM per each well using DharmaFECT (Dharmacon–Thermo Scientific) according to the manufacturer’s protocol. The cells were incubated for 64 h at 37 °C in a CO_2_ incubator. Gene expression knockdown was verified by western blotting.

### Generation of patient iPS cell-derived neuronal progenitors

All iPS derived neural progenitors were provided by Pfizer Neuroscience (Cambridge, MA, USA). The iPS cell lines D2 and D3 and family control cell line C1 were generated using a non-integrated approach by Dr Hongjun Song at Johns Hopkins University and cultured as described previously.^8^ The Detroit iPS cell line was generated by Dr S. Engle at Pfizer Neuroscience. Detroit 551 fibroblasts were obtained from ATCC (CCL-110) and maintained in Knockout DMEM (Life Technologies 10829-018), 15% FBS (Life Technologies), 1x Non-essential amino acids (Life Technologies 11140-050), 2 mM L-glutamine (Life Technologies 25030-081), 1x Gentamicin (Life Technologies 15710-072), 0.1 mM β-mercaptoethanol (Sigma M-7522). The cells were reprogrammed with individual lentiviruses expressing c-MYC, KLF4, SOX2, OCT3/4, NANOG, and LIN28. One colony, Detroit 551-3, with ES-like morphology, high expression of pluripotency markers (for example, OCT3/4, TRA-1-60, TRA-1-81, SSEA4), a normal karyotype, a normal karyotype and the ability to form all three germ layers in a teratoma assay was used in all subsequent experiments. The cells were maintained on mitomycin C (Sigma M0503)-treated mouse embryonic fibroblasts (MEF) and grown in hPSC media [DMEM/F12 (Life Technologies 11330-057), 20% knockout serum replacement (Life Technologies, 10828-028), 2 mM L-glutamine (Life Technologies 25030-081), 1x Gentamicin (Life Technologies 15710-072), 0.1 mM β-mercaptoethanol (Sigma M-7522), FGF2 (Life Technologies PHG0263)]. Cells were enzymatically passaged using 1 mg ml^–1^ Dispase (Life Technologies 17105-0410) to every 4–6 days to fresh MEF feeder cells.

Neuronal progenitor differentiation from iPS cell lines was performed as described previously.^8^ The resulting neuronal progenitors were seeded in six-wells plates pre-coated with 20 μg ml^–1^ poly-L-ornithine hydrobromide (Sigma P3655)/5 μg ml^–1^ natural mouse laminin (Life Technologies 23017-015)) and maintained in NP media [50% Neurobasal Media (Life Technologies 21103-0490), 50% DMEM/F12 (Life Technologies 10565-018), 0.5 × N2, 0.5 × B27 (Life Technologies 17504-044), 1 mM L-glutamine (Life Technologies 25030-081), 1x Gentamicin (Life Technologies 15710-072), 10 ng ml^–1^ EGF (R&D Systems 236-EG), 10 ng ml^–1^ FGF2 (Invitrogen PHG0263)]. Cells were passaged by aspirating the media and washing three times with PBS. One milliliter of Accutase (Invitrogen A1110501) was used to gently detach the cells. Equal volume of DMEM-F12 media was added to neutralize the enzyme activity and the cells were collected by gently pipetting the suspension and centrifuged at 1000 r.p.m. for 5 min. Supernatant was disposed and the cell pellet was resuspended in NP media supplemented with 10 μM Rock inhibitor (Y-27632, Sigma) and plated in 1:3 ratio on new pre-coated plates.

### Immunoprecipitation and western blot

Briefly, detergent-soluble proteins were isolated from cells by disruption in lysis buffer 20 mM Tris-HCl (pH 7.5), 150 mM NaCl, 1 mM Na_2_EDTA, 1 mM EGTA, 1% triton, 2.5 mM sodium pyrophosphate, 1 mM β-glycerophosphate, 1 mM Na_3_VO_4_, 1 μg ml^–1^ leupeptin, 10 mM DTT with Roche protease inhibitor, phosSTOP (Roche, 04906837001). Detergent-insoluble proteins were removed by centrifugation at 10 000 *g* for 10 min, and the soluble fraction was retained. Cell lysates were adjusted to equal protein amount (1 μg μl^–1^) using cell lysis buffer. Cell lysates normalized for the protein concentration were incubated with mouse IgG (Millipore, 12487) Protein A agarose beads for 2 h at 4 °C. Agarose beads were collected after a brief centrifugation and supernatant is collected and added with tagged agarose (Sigma, A2095) washed three times in cell lysis buffer and the final volume is adjusted to 750 μl and incubated overnight at 4 °C. The beads were precipitated and washed three times to remove non-specifically bound proteins. Immune complexes are eluted from the beads by boiling them in SDS loading buffer (10% SDS, 300 mM Tris-HCl, pH 7.2, 0.05% bromophenol blue, 50% glycerol, 10% β-mercaptoethanol). The complexes were analyzed on SDS PAGE. Tagged agarose beads used in the present study include: Flag (Sigma, A2220), HA (Sigma, A2095), V5 (Abcam, ab1229).

Antibodies used in this study are detailed in Supplementary Table 1. As there is some ambiguity concerning DISC1 isoforms and antibody recognition of specific isoforms, we utilized a robustly characterized antibody to the 100 Kilodalton (KD) form of DISC1 described in refs 15 and 16. Importantly, these studies showed a diminution of the immuno-reactivity of the 100 KD form of DISC1 following genetic silencing of this isoform. Western blotting was performed using the NuPAGE system (Invitrogen) according to the manufacturer’s instructions. Briefly cell lysates were boiled in SDS loading buffer and loaded on to NuPAGE precast 4–12% gels in MOPS buffer along with pre-stained protein marker (Bio-Rad) and resolved at 120 V. The resolved proteins were then electrotransfered onto nitrocellulose membranes (Whatman) in 1X transfer buffer with 20% methanol for 2 h at 25 V or overnight at 11 V. The membrane was then blocked in 5% (w/v) non-fat dry milk (Marvel) in TBST (25 mM Tris-HCl; pH 7.6, 100 mM NaCl, 0.5% Tween 20) for 1 h at room temperature. Membranes were then probed with specific primary antibodies at appropriate dilutions in 1% (w/v) marvel/TBST solution and incubated for 2 h at room temperature or overnight at 4 °C. The membranes were washed three times for 15 min each in TBST. Membranes are then probed with the corresponding secondary antibody diluted in 1% milk/TBST solution for 1 h at room temperature. Membranes were then washed three times for 10 min. Chemiluminescence (ECL) western blotting substrate (Thermo Scientific) was used for detecting the HRP conjugates on the membrane. Chemiluminescent images of immune detected bands were recorded on blue-light sensitive autoradiography X-ray films, which were then developed using the Kodak X-Omat Model 2000 processor and band intensities were quantified using image J. Alternatively blots were visualized using the Licor Odyssey system and quantified using LICOR Image Studio Lite (Licor Biosciences).

### Peptide array

Peptide libraries were produced by automatic SPOT synthesis and synthesized on continuous cellulose membrane supports on Whatman 50 cellulose membranes using Fmoc-chemistry with the AutoSpot-Robot ASS 222 (Intavis Bioanalytical Instruments, Köln, Germany) as previously described.^17^ Phosphorylation of peptide array peptides by constitutively active GSK3 (14-306, Millipore UK) was undertaken as previously described.^18^ Phosphorylation was detected using a phospho-serine specific antibody (ab9332, Abcam, UK).

### Protein expression and purification

Amino-terminal His6 tagged human FBXW7 (residues 263–707) and truncated Skp1 (Schulman *et al.*, 2000) were co-expressed as a dicistronic message in *Escherichia coli*. The Skp1-FBXW7 complexes were purified by Ni2+-nitrilotriacetic acid (NTA) affinity chromatography followed by tobacco eth virus (TEV) protease cleavage of the His6 tag, and by anion exchange and gel filtration chromatography. For crystallization, the Skp1-FBXW7 complex was concentrated to 48 mg ml^–1^ by ultrafiltration in 20 mM HEPES-Na (pH 7.4), 200 mM NaCl and 5 mM dithiothreitol (DTT). The DISC1 peptides were synthesized chemically and purified by HPLC.

### Crystallization and structure determination

The Skp1-FBXW7-DISC1 complexes were prepared by mixing a 48 mg ml^–1^ solution of Skp1-FBXW7 with a twofold molar excess of the DISC1 peptide (residues 193–207, phosphorylated at Thr198 and Ser202) in 25 mM Tris-HCl (pH 8.0), 200 mM NaCl, and 5 mM DTT. The Skp1-Fbw7-DISC1 peptide complex was crystallized from 100 mM HEPES-Na (pH 7.4) and 1.2 M Li_2_SO_4_ by the hanging-drop vapor diffusion method at 4 °C. Crystals were flash-frozen in solutions containing saturated Li_2_SO_4_. Diffraction data were collected at the X29 beamline of the National Synchrotron Light Source (NSLS). Data were processed using the HKL2000 suite. All crystals contain one complex in the asymmetric unit. The structures were determined by molecular replacement with the program MOLREP of the CCP4 suite^19^ and the Skp1-FBXW7 structure^19^ was used as the search model. The DISC1 peptide was built using 2Fo—Fc and Fo—Fc maps with Coot^20^ and refined using REFMAC5.^21^ Residues 204–207 of DISC1 are not visible in the electron density maps, and presumably disordered.

### Isothermal titration calorimetry

All measurements were performed at 25 °C with a NANO ITC System (TA Instruments, New Castle, DE, USA). Protein samples were dialyzed against a solution containing 20 mM HEPES-Na (pH 7.4), 200 mM NaCl, and 2 mM β-mercaptoethanol. Typically, the concentrations of Skp1-FBXW7 and DISC1 peptides were 50 mM and 1 mM, respectively. Titration data were analyzed using the TA Nano Analyze software, and the reported dissociation constants and their standard deviations were derived from two to three independent measurements.

### Data collection and statistics

All experiments were replicated at least three times as described in the figure legend. Statistical analyses used for comparison are reported in each figure legend. Power calculations were undertaken to evaluate appropriate samples sizes for each experimental procedure.

## Results

### The Ubiquitin-Proteosome System regulates DISC1 turnover

**Figure 1:**
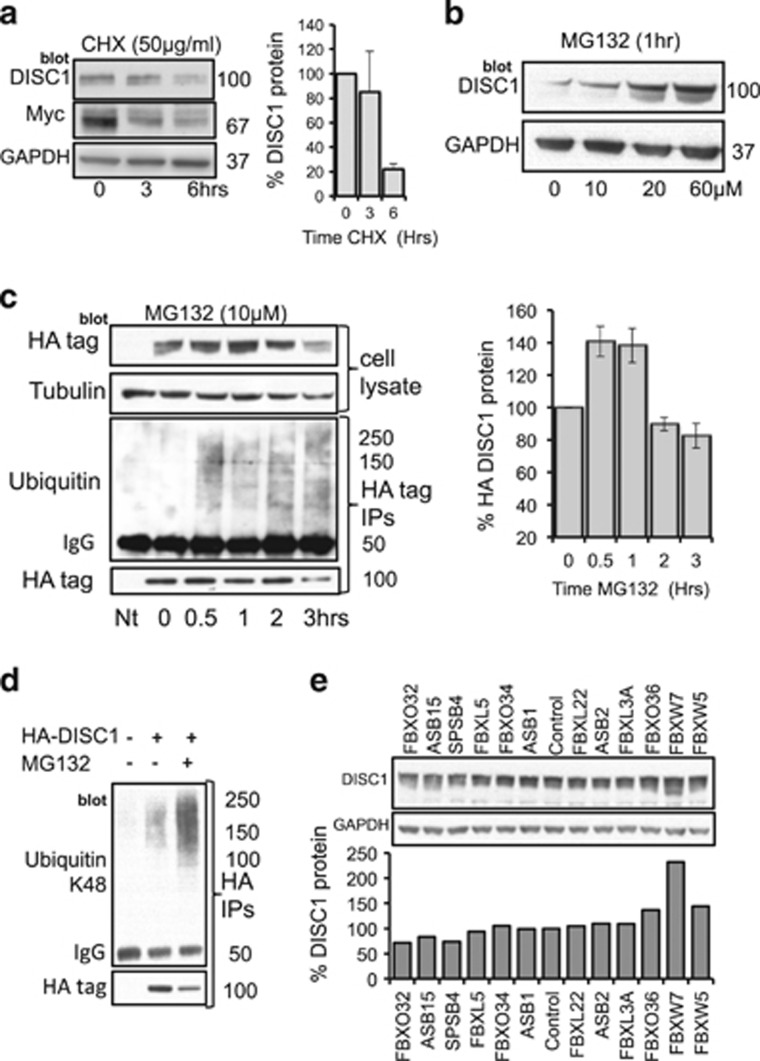
DISC1 turnover is regulated by the ubiquitin-proteosome system. (**a**) HEK293 cells were treated with cycloheximide (50 μg ml^–1^) for indicated times before cellular lysates were isolated and probed for DISC1 and another known FBXW7-substrate Myc. Levels of DISC1 protein were evaluated by densitometry (bar-chart, *n*=3) (**b**) HEK293 cells were treated with indicated concentrations of MG132 (10–60 μM) for 1 h before cellular lysates were isolated and probed for DISC1. (**c**) HEK293 cells were transfected with HA-DISC1 and treated with MG132 (10 μM) for indicated times before cellular lysates were prepared (upper panels) and blotted for HA and Tubulin. HA-DISC1 levels were evaluated using densitometry (bar-chart, *n*=3). ‘Nt’ indicates no treatment control. The HA-tag was immunoprecipitated and blotted for HA and ubiquitin (lower panels). (**d**) HEK293 cells were transfected with HA-DISC1 and treated with MG132 (10 μM) for 1 h before cellular lysates were prepared and HA-tag immunoprecipitated and blotted for HA (lower panel) and ubiquitin chains linked specifically via lysine 48 (upper panel). (**e**) A siRNA library against human F-Box proteins was transfected individually into HEK293 cells, before cellular lysates were recovered and blotted for endogenous DISC1 and GAPDH. DISC1 levels were evaluated by densitometry (bar-chart, *n*=1).

Endogenous protein levels of DISC1 in HEK293 cells are rapidly decreased following cycloheximide treatment (Figure 1a) and elevated after application of the proteosome inhibitor MG132 (Figure 1b), suggesting that DISC1 turnover is regulated by the UPS. Exogenously HA-tagged DISC1 protein expression is transiently increased following MG132 treatment and HA-DISC1 immunoprecipitates co-purify with ubiquitin after a 30 min exposure to MG132 (Figure 1c). Antibodies specific for K48-linked chains recognized the ubiquitin associated with DISC1 immunoprecipitates, further supporting the notion that the UPS regulates DISC1 stability (Figure 1d). Indeed, DISC1 ubiquitination can be amplified by over-expression of either DISC1 or ubiquitin in a manner that was not enhanced further by MG132 (Supplementary Figure 1a).

Mass spectrometry following excision of DISC1 protein bands allowed us to identify the predominant ubiquitination site on DISC1 as lysine 372 (K372) (Supplementary Figure 1b). However, that ubiquitination was still evident in a K372R mutant, albeit occurring at a significantly lower level when compared with wild-type (Supplementary Figure 2), suggests that there may be additional ubiquitination site(s) on DISC1. Although it may be that such site(s) are cryptic and only evident when ubiquitination is ablated at the major lysine 372 site through mutation.

To identify the E3 ligase responsible for DISC1 ubiquitination we utilized a siRNA library against all known human F-Box proteins (see Materials and Methods). This allowed us to demonstrate that silencing of FBXW7 specifically and robustly increased protein levels of DISC1 in HEK293 cells (Figure 1e and Supplementary Figure 3B). MYC, a well-characterized substrate for FBXW7 exhibited a similar pattern of upregulation to DISC1 (Supplementary Figure 3B), adding another level of validation to the notion that DISC1 is an FBXW7 substrate.

### FBXW7 binds directly to DISC1 to regulate turnover

**Figure 2:**
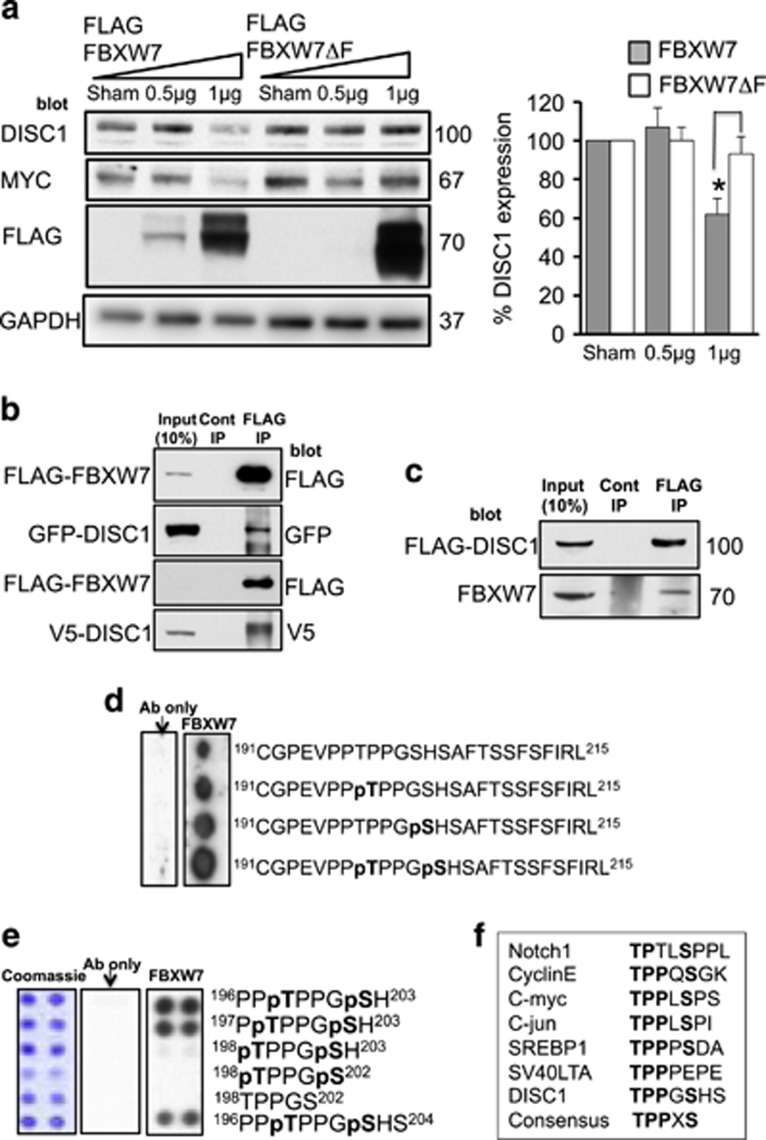
FBXW7 binds DISC1 via a PD motif to regulate turnover of DISC1. (**a**) HEK293 cells were transfected with FLAG-tagged FBXW7 or FLAG-tagged FBXW7 without the F-Box substrate-binding domain (Δ-F). Lysates were prepared and blotted for DISC1, c-MYC, FLAG and GAPDH. Relative levels of DISC1 expression were calculated (right hand panel). *=*P*<0.05 as measured by Student’s *t*-test, *n*=3 (**b**) HEK293 cells were transfected with FLAG-FBXW7 and GFP-DISC1 (upper panels) or FLAG-FBXW7 and V5-DISC1 (lower panels). FLAG was immunoprecipitated from cell lysates and blotted for FLAG, GFP and V5. (**c**) HEK293 cells were transfected with FLAG-DISC1. FLAG was immunoprecipitated from cell lysates and blotted for FLAG and endogenous FBXW7. (**d**) and (**e**) Peptide arrays of sequences containing the DISC1 PD were overlayed with a purified protein complex containing 6His-FBXW7 and Skp1. Arrays were then probed for His tag. (**f**) Sequences of known FBXW7 substrates contain a PD that is also present in DISC1.

To verify FBXW7 as the F-Box ligase for DISC1, we transiently overexpressed either wild-type FBXW7 or a mutant FBXW7 that was devoid of the F-Box domain (meaning that it could not interact with other members of the E3 ligase complex) and evaluated endogenous levels of both DISC1 and MYC, a known FBXW7 substrate (Figure 2a). Both DISC1 and MYC protein levels were significantly decreased following expression of active FBXW7 but not the mutant.

DISC1 half-life following cycloheximide treatment was also shortened in cells expressing recombinant FBXW7 (Supplementary Figure 3A). Furthermore, FBXW7 and DISC1 exist together in a protein complex in cells, being co-immunoprecipitated from cells co-expressing FLAG-FBXW7 with either GFP-DISC1 or V5-DISC1 (Figure 2b). Furthermore, immunoprecipitates of FLAG-DISC1 from transfected cells contained endogenously expressed FBXW7 (Figure 2c).

**Figure 3:**
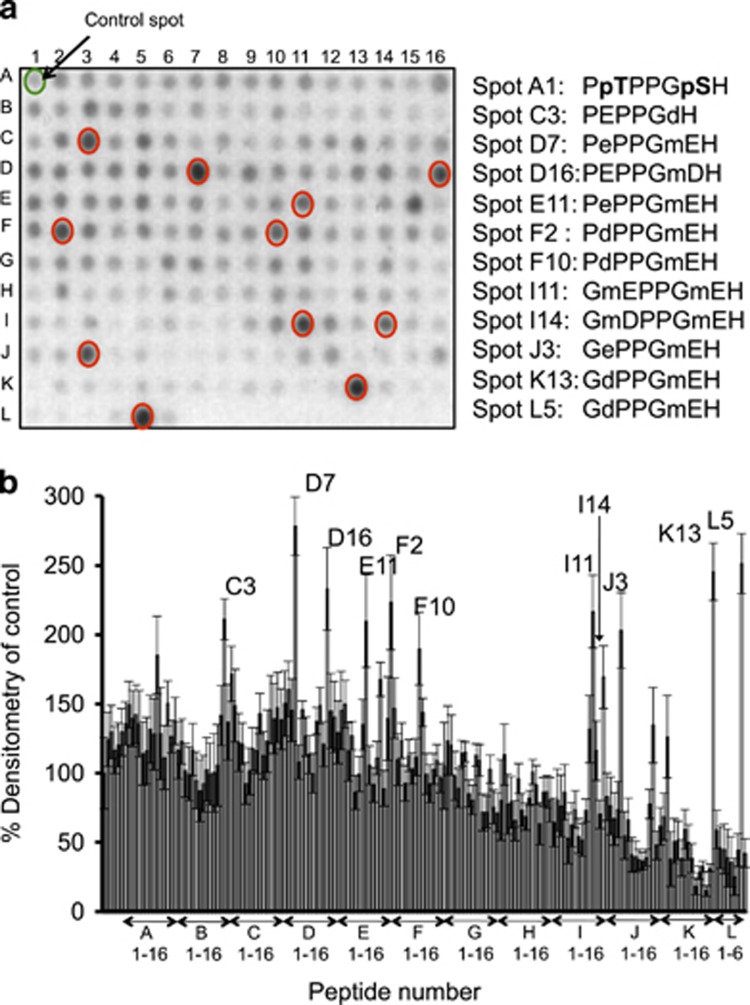
Substitution analysis pinpoints novel binding motifs for FBXW7. (**a**) Peptide array grids containing substitution of residues within the core binding motif domain ^197^PTPPGSH^202^ (with a range of native and non-native residues) were overlaid with a purified protein complex containing 6His-FBXW7 and Skp1. mD and mE are *N*-methyl aspartic acid and *N*-methyl glutamic acids respectively. (**d**) and (**e**) are the D-forms of aspartic acid and glutamic acid. (**b**) Arrays were then probed for 6His and spots that were over 200% of the control identified by densitometry.

**Figure 4:**
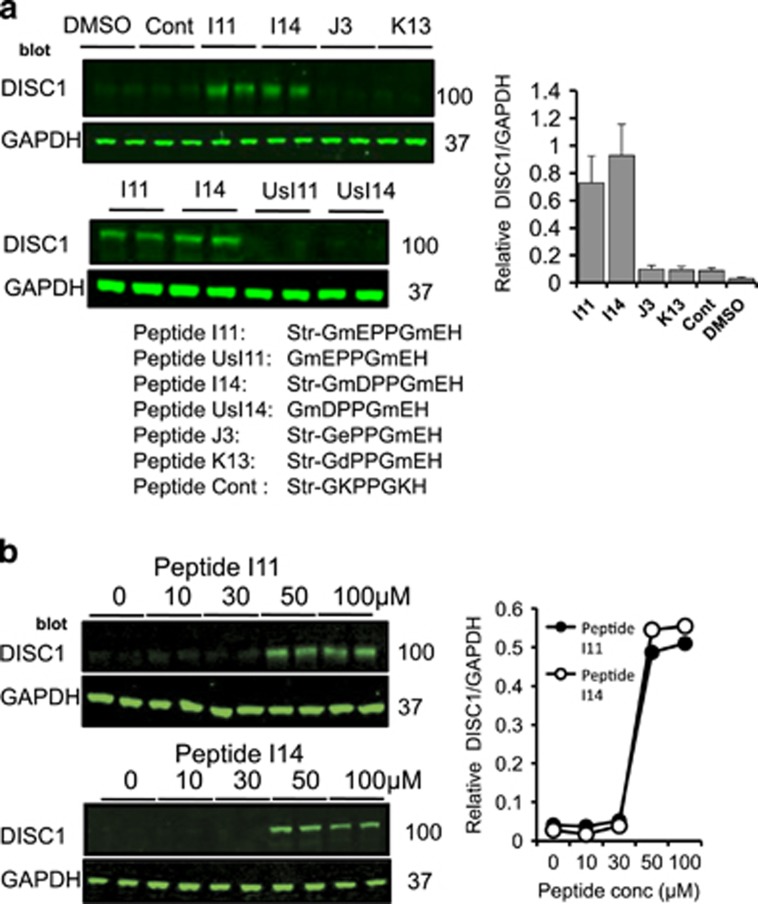
Disruption of the FBXW7/DISC1 complex using cell-permeable peptide analogs of the DISC1 PD upregulate DISC1 protein levels in HEK293 cells. (**a**) HEK293 cells were treated with the indicated peptides (sequences delineated below Figure 4a) for 3 h (100 μM) before cellular lysates were prepared and blotted for DISC1. ‘Str’ represents stearoylated peptides and ‘Us’ represents unstearoylated peptides. (**b**) HEK293 cells were treated with the indicated concentrations of peptides I11 and I14 for 3 h before cellular lysates were prepared and blotted for DISC1.

As FBXW7 binds to PD motifs on substrates that contain the consensus ‘TPPxS’ (Figure 2f),^22^ we undertook sequence analysis of the DISC1 sequence and identified a candidate PD motif, ^198^TPPGS^202^. Using peptide array technology, a technique that allows interrogation of a library of immobilized peptides,^23^ we confirmed the phosphorylation-driven interaction between peptides containing the DISC1 PD motif and a purified protein complex of Skp1-FBXW7 (Figure 2d). Inclusion of phosphorylated versions of the residues ^198^T and S^202^ within the PD domain dramatically increased the association of the FBXW7 complex when compared with mono-phosphorylated or unphosphorylated peptides (Figure 2d). Truncation analysis allowed us to delineate the minimum core binding sequence within the PD domain as ^197^PTPPGSH^203^ (Figure 2e) and this peptide was used as a starting point to develop a protein-protein interaction disruptor peptide for the DISC1-FBXW7 complex. Rational rounds of peptide substitution at non-essential positions 1, 4 and 7, in order to increase affinity, and at positions 2 and 6, to avoid the need for labile phospho-groups within the PTPPGSH sequence, allowed us to identify 11 peptides that exhibited more than a twofold increase in binding potential for the FBXW7 complex, compared to the dually-phosphorylated control sequence (Figure 3). All 11 had N-methyl glutamic acid at position 6. Four of the identified peptides (I11, I14, J3 and K13) were modified by addition of a stearate group at the N-terminus to allow peptides to cross the plasma membrane (a technique used successfully previously by us and others^24, 25, 26, 27^) to test their ability to affect the cellular protein expression of endogenous DISC1 in HEK293 cells (Figure 4). Peptides I11 and I14 (but not the unstearoylated versions of I11 and I14) robustly and significantly increased DISC1 protein levels whereas peptides J3, K13 and a control peptide, where the acidic residues had been replaced with basic ones, did not (Figure 4a). The predominant DISC1 band detected was the 100 KD species (Supplementary Figure 10). The reasons behind the success of stearoylated peptides I11 and I14 in upregulating DISC1 compared with the failure of stearoylated versions of J3 and K13 remain unclear as all four peptides showed similar binding on peptide array. It is possible, however, that the N-methylation at position 2 confers resistance to peptidases. DISC1 upregulation was successful at doses over 50μM (Figure 4b) but were relatively short lived due to peptide instability (Supplementary Figure 4A). Improvements to the stability, potency and cell permeability of the peptide may be achieved by stabilizing alpha helices using novel peptide stapling technology.^28, 29^Interestingly, the protein levels of other previously identified FBXW7 substrates (Notch1, c-Myc, c-Jun and cyclin E)^22^ were not affected by treatment with peptides I11 or I14 (Supplementary Figure 4B), suggesting that the disruptor peptides were selective for the DISC1-FBXW7 interaction (see Discussion section).

### Phosphorylation of DISC1 at Thr198 and Ser202 is required for FBXW7 binding.

**Figure 5:**
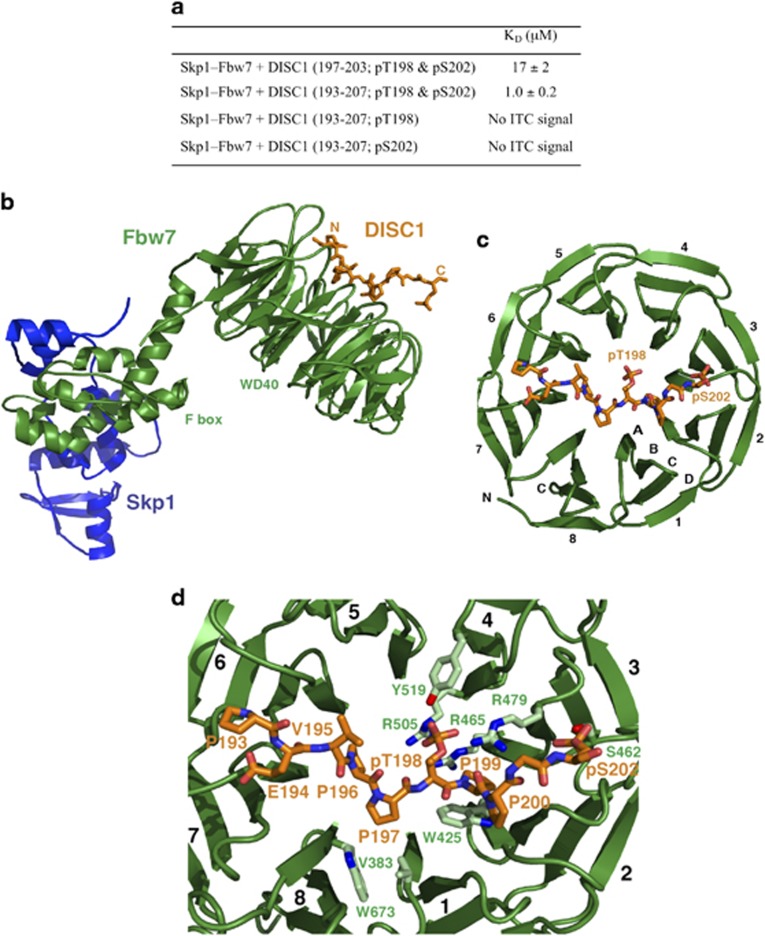
Structure and interaction of the Skp1-FBXW7-DISC1 PD complex. (**a**) Dissociation constants determined by ITC for different DISC1 PD peptides. (**b**) Overall architecture of the complex. Skp1, FBXW7, and DISC1 are in blue, green, and orange, respectively. (**c**) Binding of the DISC1 peptide at the top face of the WD40 domain of FBXW7. The eight FBXW7 blades and the strands for one blade are labeled. (**d**) Close-up view of the DISC1-FBXW7 interface showing interacting amino acids of FBXW7 (light green) and DISC1 (orange).

To test whether phosphorylation of both the Thr198 and Ser202 sites in DISC1 is required for binding to FBXW7, we used isothermal titration calorimetry (ITC) to measure the binding affinity of various phosphorylated DISC1 peptides to the Skp1-FBXW7 complex (Supplementary Figure 5). The 7- and 15-residue core peptides of DISC1 phosphorylated at Thr198 and Ser202 (denoted DISC1^197–203/pT198/pS202^ and DISC1^193–207/pT198/pS202^) bind to Skp1-FBXW7 with dissociation constants (K_D_) of 17±2 μM and 1.0±0.2 μM, respectively (Figure 5a). By contrast, neither of the singly phosphorylated DISC1^193–207/pT198^ and DISC1^193–207/pS202^ peptides gave a measurable enthalpy signal (Figure 5a). Thus, specific interaction of DISC1 with the Skp1-FBXW7 complex requires simultaneous phosphorylation at Thr198 and Ser202 within the DISC1 PD motif.

### Identification of GSK3 as a putative kinase for the DISC1 phosphodegron

To identify putative kinases responsible for phosphorylation of residues T198 and S202 within the DISC1 phosphodegron were used an *in silico* method using a group based prediction system (iGPS, Cuckoo workgroup, v1.0).^30^ This algorithm uses known consensus motifs to assign putative kinases for residues within a sequence of interest, combined with protein-protein interaction data as a major contextual factor to identify potential false-positives. This analysis identified glycogen synthase kinase-3 (GSK3) as the putative kinase responsible for DISC1 phosphorylation at both T198 and S202 (Supplementary Table 2), this is in agreement with other FBXW7 substrates where the central phospho-threonine is followed by a phospho-serine in the +4 position, which acts to prime GSK3. In agreement with this notion, active GSK3 was able to phosphorylate peptide arrays containing the DISC1 motif ^198^TPPGS^202^ (Supplementary Figure 10E). DISC1 has been previously shown to directly bind to GSK3 and regulate its function,^31, 32^ however this action may be bi-directional as GSK3 may regulate DISC1 turnover by phosphorylation of its phosphodegron.

### Crystal structure of the tertiary Skp1-FBXW7-DISC1 phosphodegron complex.

To investigate the basis for phosphorylation-dependent binding of FBXW7 to the DISC1 PD motif, the X-ray crystal structure of the Skp1-FBXW7 complex bound to DISC1^193–207/pT198/pS202^ was determined at 2.6 Å resolution (Supplementary Figure 6, deposited structure 5V4B). As anticipated, residues 193–202 of the DISC1 peptide bind to the narrow face of the FBXW7 WD40 domain extending from blades 6/7 at one side of the surface, across the central channel, to blades 2/3 at the other side (Figures 5b and c). The spatial arrangement of the DISC1 PD motif on the WD40 β-propeller closely resembles that in other Skp1-FBXW-substrate protein complexes^33, 34^ (Supplementary Figure 8). The central five-residue segment (Pro196, Pro197, pThr198, Pro199 and Pro200) of DISC1 adopts a twisted U-shaped-backbone conformation to enable dipping of the pThr198 phosphate group into the β-propeller channel. Interestingly, residues 195–197 and 199–201 immediately preceding and following pThr198 form two left-handed polyproline type II helices. As a result, the peptide chain does not enter the beta-propeller channel and stays on top of the blades, thereby facilitating pThr198 recognition by FBXW7. Moreover, the C-terminal pSer202 side chain binds outside the central channel on top of blades two and three and also plays a critical role in specifying and stabilizing the DISC1 PD motif required for FBXW7 binding (see below).

DISC1-FBXW7 binding is mediated by both hydrogen bond networks and van der Waals contacts (Figure 5d and Supplementary Figure 7). The pThr198 phosphate group tucks into a positively charged pocket at the inner rim of the FBXW7 β-propeller channel and makes charge-stabilized hydrogen bonds with the guanidinium groups of Arg465, Arg479 and Arg505, as well as the hydroxyl group of Tyr519 from blades three and four (Supplementary Figure 7). The side chain of pSer202 is oriented so as to allow its phosphate group to make hydrogen bonds with the hydroxyl group of Ser462, the backbone amide of Thr363 and the guanidinium groups of Arg441 and Arg479 on top of blades two and three (Figure 5d and Supplementary Figure 7). The three residues between pThr198 and pSer202 contribute additional contacts to FBXW7 (Figure 5d and Supplementary Figure 7). The pyrrolidine ring of Pro199 fits into a sandwich-like structure formed by the side chains of Trp425 and Arg479, while the backbone carbonyl groups of Pro199 and Pro200 hydrogen bond with the Arg479 side chain. Moreover, the DISC1 peptide preceding pThr198 packs into two partially solvent-exposed hydrophobic pockets on FBXW7. Pro196 and Pro197 engage in van der Waals interactions with Val383, Val671 and Trp673 of WD40 repeats 1 and 8, whereas Pro193, Glu194 and Val195 interact with Leu559, Leu583, Ala599 and Ala626 of WD40 repeats 5, 6 and 7. Thus, DISC1 recognition specificity is achieved by a combination of satisfaction of hydrogen bond donors and acceptors from pThr198 and pSer202 and a set of polar residues flanking the narrow face of the WD40 domain, together with stacking interactions and van der Waals contacts. The ternary Skp1-FBXW7-DISC1^193–207/pT198/pS202^ structure explains why the doubly phosphorylated pThr198/pSer202 DISC1 motif is an optimal, high-affinity degron (Figure 5a). These structural and energetic features justify exploiting specific DISC1-target site interactions at an atomic level to enable rational improvement of potential DISC1 ubiquitination and degradation inhibitors.

### Disruption of the FBXW7-DISC1 complex restores DISC1 protein levels in a human cellular model of psychiatric disease

DISC1 protein levels are known to be reduced in lymphoblastoid cells from subjects carrying the *t*(1;11) DISC1 translocation^35^ and iPS derived neurons from a DISC1 frameshift mutation,^9^ although in the latter case, the causality of this mutation remains unclear based on pedigree size and identification of the mutation in control subjects.^36^ We utilized human neuroprogenitor cells (NP cells), differentiated from patient derived induced pluripotent stem cells (iPS cells) from the latter genotype^8, 37^ to determine whether disruption of the FBXW7-DISC1 complex could restore DISC1 protein expression to control levels. These cells have previously been characterized by Wen *et al.*^9^ and exhibit a single DISC1 band at 100 Kilodaltons.

**Figure 6:**
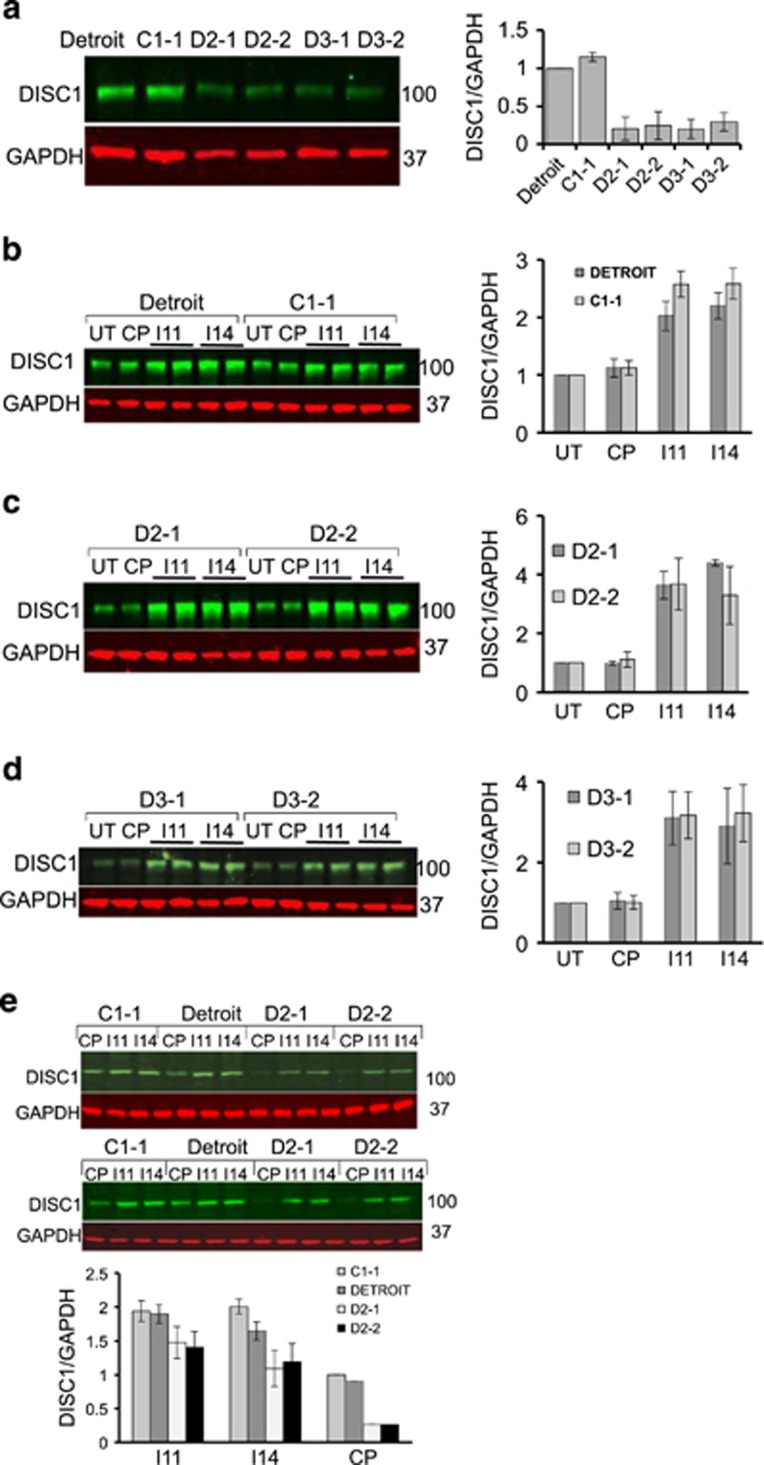
DISC1-FBXW7 disruptor peptides restore DISC1 protein expression in neural progenitors derived from patient iPS cells carrying a DISC1 mutation. (**a**) Relative DISC1 levels were determined in cellular lysates from neural progenitors derived from different iPS cell lines as described previously.^8^ (**b**) Neural progenitors derived from control subjects were treated with the indicated peptides (100 μM) for 3 h prior to cellular lysate being isolated and blotted for DISC1 and GAPDH. Relative DISC1 levels were calculated by densitometry (*n*=3). (**c** and **d**) Neural progenitors derived from patients carrying a 4-bp deletion in the *DISC1* gene were treated with the indicated peptides (100 μM) for 3 h prior to cellular lysate being isolated and blotted for DISC1 and GAPDH. Relative DISC1 levels were calculated by densitometry (*n*=3). (**e**) Neural progenitors derived from control subjects and patients carrying a 4-bp deletion in the *DISC1* gene were treated with the indicated peptides (100 μM) for 3 h prior to cellular lysate being isolated and blotted for DISC1 and GAPDH. Relative DISC1 levels were calculated by densitometry (*n*=3).

In agreement with previous work,^9^ the 100 KD form of DISC1 protein (Supplementary Figure 10) levels in cells derived from control subjects were significantly higher than in cells derived from subjects carrying the DISC1 frameshift mutation (Figure 6a). We could detect the complex between DISC1 and FBXW7 endogenously in human iPS derived neural progenitors (Supplementary Figure 10C) and the comparatively low levels of DISC1 in patient-derived cells could be enhanced by MG132 treatment suggesting involvement of the UPS (Supplementary Figure 10D). Crucially, we demonstrate that incubation of WT DISC1 NP cells with stearoylated peptides I11 and I14 resulted in a marked increase in endogenous DISC1 levels, suggesting that DISC1 can be regulated via the action of FBXW7 in these cells (Figure 6b). Pleasingly, application of the I11 and I14 peptides to the frameshift DISC1 patient-derived cells also significantly increased DISC1 protein levels (Figures 6c and d). Moreover, further analysis revealed that peptides I11 and I14 (but not control peptide) enhanced levels of DISC1 in NP cells derived from psychiatric disease patients to that of control subjects (Figure 6e).

## Discussion

DISC1 is a vital intracellular scaffold protein that orchestrates neural cell differentiation and signaling,^38^ hence the fine control of DISC1 stability/turnover is fundamental for the maintenance of brain function. In our efforts to investigate the factors regulating DISC1 stability, we discovered that DISC1 turnover is regulated by the UPS, but more specifically that FBXW7, the substrate recruiting subunit of the Cullin-RING E3 ligase, orchestrates ubiquitination to control protein levels of DISC1.

The half-life of DISC1 has been previously calculated at around 6 h and this can be increased following blockade of the UPS,^39^ suggesting that DISC1 can be modified by ubiquitin prior to its proteolysis by the 26S proteosome. Indeed, we confirm that DISC1 can be ubiquitinated on lysine 372 and that the ubiquitin chains are linked at K48 (Figure 1), a requirement for high-affinity binding of the proteolytic substrate to the regulatory particle of the 26S proteosome.^40^ To explore the molecular mechanisms underlying DISC1 ubiquitination, we utilized a siRNA screen (Figure 1) to identify FBXW7, an F-box protein subunit of an (Skp, Cullin, F-box) SCF-type cullin-RING ubiquitin ligase complex,^41^ that functions as a binding protein for DISC1. Other previously discovered substrates of FBXW7 include proto-oncogene protein products such as cyclin E, c-Myc and c-Jun,^22^ that mediate forward momentum through the cell cycle. Interestingly, they also include Notch, a protein that is a key regulator of neuronal differentiation, migration and synaptic plasticity.^42^ All of these FBXW7 substrates contain a characteristic TPPxS motif in which conserved Thr and Ser residues are phosphorylated for their recognition by the SCF complex.^22, 34^ We demonstrate that double phosphorylation of the DISC1 degron is essential to provide sufficient affinity for FBXW7 binding. Our crystallographic analysis reveals that the phosphate groups of the DISC1 pThr198 and pSer202 residues interact with two phosphate-binding sites on the WD40 domain of FBXW7 in a manner analogous to those of cyclin E^34^ (Supplementary Figure 8). The cyclin E degron forms a polyproline helix so as to enable binding of two phosphate groups to the FBXW7 pockets, whereas DISC1 utilizes two polyproline helical segments to promote interaction with FBXW7. Moreover, hydrophobic interactions also contribute to the stable association of the FBXW7-substrate complexes. Our ITC data have shown that removing the four mostly hydrophobic residues (Pro193, Glu194, Val195 and Pro196), preceding pThr198, lowers the binding affinity of DISC1 to FBWX7 by 17-fold; these four residues form a more extended conformation across the top of blades 5, 6 and 7, while the corresponding residues in cyclin E adopt a β turn conformation. Our structural studies provide a solid basis for future structure-based drug discovery efforts to develop ligands that inhibit DISC1 degradation.

Studies with peptides derived from protein-protein interaction interfaces can usefully allow both definition of the key contributory binding determinants to such interactions as well as deconvolution of the specific interactions that orchestrate defined downstream signaling events. Indeed, we have used this technique extensively in the past to characterize novel functions for proteins such as ARHGAP21,^27^ PDE8A^25^ and PDE4D,^24, 26^ amongst others. Using modified versions of the minimum core sequence of the DISC1 PD motif conjugated to a membrane-permeabilising stearoyl group, we were able to upregulate DISC1 expression in a human cell line that expressed low levels of DISC1 protein (Figure 4). We were also able to rescue the depleted DISC1 protein levels observed in the same iPS cell-derived neural progenitors from patients with a frameshift mutation of DISC1 that is known to lead to pre-synaptic deficits (Figure 6).^9^ Importantly, our peptide restored DISC1 in patient cells back to control levels (Figure 6e) and not to a super-elevated DISC1 protein concentration, which may lead to DISC1 aggregation with associated physiological consequences to mitochondrial trafficking,^43^by disruption of binding to nuclear distribution element 1 (NDEL1)^44^ or the dopamine transporter^45^ It has been suggested that the mutant DISC1 protein produced by the frameshift (mDISC1) interacts with wild-type DISC1 (wDISC1) to form aggregates that both deplete the soluble pool of wDISC1 and promote its ubiquitination.^9^ This mechanism has been proposed as an alternative to haploinsufficiency to explain why so little DISC1 protein is expressed in these cells, however little insight has been offered into the molecular events that underpin this hypothesis. We confirm that DISC1 can be ubiquitinated and that FBXW7 is the likely E3 ligase that facilitates this modification.

Our findings raise the possibility that disruption of FBXW7-DISC1 complex could be considered as a novel therapeutic strategy to combat reduced DISC1 levels that stem from mutations resulting in DISC1 haploinsufficiency.^1, 7^ However, the relevance of DISC1 in sporadic chronic mental disease has yet to be fully elucidated. A recent small study demonstrated that diminished peripheral DISC1 protein levels are a disease trait marker associated with sporadic schizophrenia and, interestingly, with smokers even in the absence of psychosis,^10^ suggesting that future opportunities may exist for an agent that selectively increases endogenous DISC1 protein levels. Global inhibition of FBXW7 would undoubtedly be deleterious as a number of cancers arise following loss of FBXW7 function via hemizygous or homozygous mutations in the *FBXW7* gene.^46^ Recently, however, the role of FBXW7 as a universal tumor suppressor was challenged by the discovery that FBXW7 is essential for B-cell survival, acting as a pro-survival factor in multiple myeloma via its regulation of NF-κB protein levels.^47^ The ability of our peptide disruptors to act specifically against the FBXW7-DISC1 interaction but not the entire FBXW7-interactome highlights the importance of the unique substrate side chain and backbone conformations in FBXW7 recognition. The selectivity could be a function of the relative affinities for the intact protein partners of FBXW7, as cyclin E exhibits significantly higher affinity than DISC1, meaning that the affinity and intracellular concentration achieved with the cell-permeable peptides conspire only to perturb the binding of DISC1. Another explanation may center on the oligomerisation status of FBXW7. It is known that that cyclin E exhibits two PD sites—a higher affinity, dually-phosphorylated C-terminal site and a lower affinity, singly phosphorylated N-terminal site.^19^ A dimeric SCF model has been suggested that relies on the presence of both PD sites, which cooperate to promote the binding and ubiquitination of cyclin E.^19^ It is possible that the sequence of DISC1 is such that it cannot bind to a dimeric SCF assembly but requires a monomeric SCF for ubiquitination. It is also noteworthy that two FBXW7 arginines (R479 and R441) that flank the phospho-Ser binding site are only loosely engaged with the pSer on DISC1. The distances are 4.3 and 4.9 Å between the phosphate and relevant atoms on R479/R441, meaning that the pSer engagement is altogether looser than the pThr engagement. It could be that a dimeric SCF assembly might cause some tightening of the R479/R441 clamp on the pSer. If that were the case, then it might contribute to differential affinities between DISC1 and other FBXW7 partner proteins (with otherwise similar PDs), and that in turn might provide a rationalization for the selectivity of the DISC1-derived peptides (based on relative affinities).

The general binding mode exhibited by the DISC1 peptide in complex with FBXW7, where the peptide binds across the ‘BC-loop face’ of the propeller (defined in^48^), is well precedented amongst β-propeller proteins. For example, the 7-bladed β-propeller, WDR5, hosts a number of proteins in such a manner, including histone H3,^49^ SET family lysine methyltransferases such as Mixed Lineage Leukemia Protein-1 (MLL1)^50^ and components of the non-specific lethal complex.^51^ In each of these cases the substrate protein binds with a short linear peptide sequence of some 6–10 residues dipping into the β-propeller canal funnel to provide focal engagement. The propeller face on either side of the canal funnel is sufficiently accommodating to allow a range of partner proteins to bind, thereby establishing multiplicity within the WDR5 interactome. FBXW7 appears to be similarly adapted, albeit with an expanded propeller structure (8 blades) and a different set of focal molecular interactions and partner proteins.^52^

DISC1 represents a key molecular hub underpinning a number of fundamental cellular functions and although peptides do not readily pass the blood-brain barrier, our cell-permeable peptides provide powerful tools to further elucidate the DISC1 biology underpinning neurodevelopment and psychosis and may open new routes to provide substrate-specific FBXW7 inhibitors for therapeutic use.
