# Supplementary figures and images for: FBXW7 regulates DISC1 stability via the ubiquitin-proteosome system
Source: Mol Psychiatry. 2017 Jul 20;23(5):1278–86. doi: 10.1038/mp.2017.138 (PMC5984089; doi:10.1038/mp.2017.138)

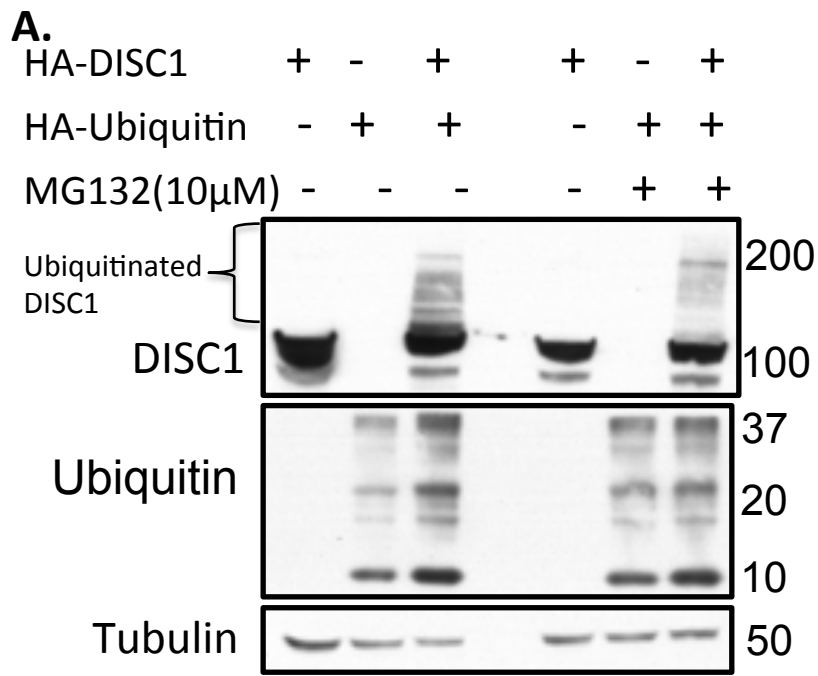

**C.**

| Lysine | Peptide               |
|--------|-----------------------|
| K372   | LQEDAVENDDDYDKAETLQQR |
| K45    | LGSVKEDYNR            |
| K18    | SLNLSLKEITTK          |
| K13    | SLNLSLKEITTK          |

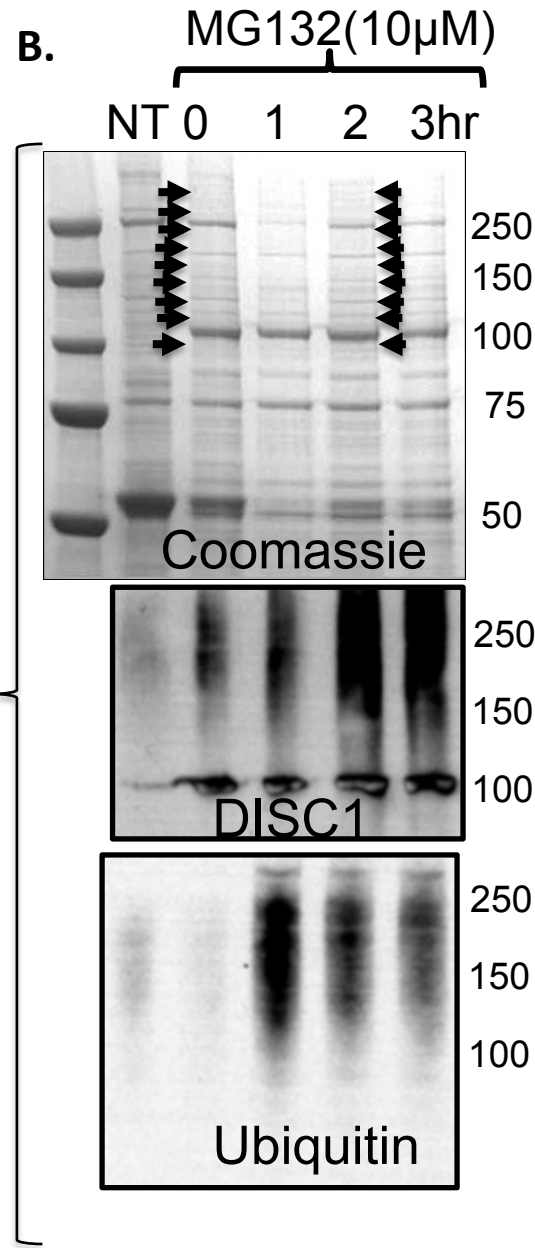

Supplement: Supplementary Figure 1 [file mp2017138x1.pdf]

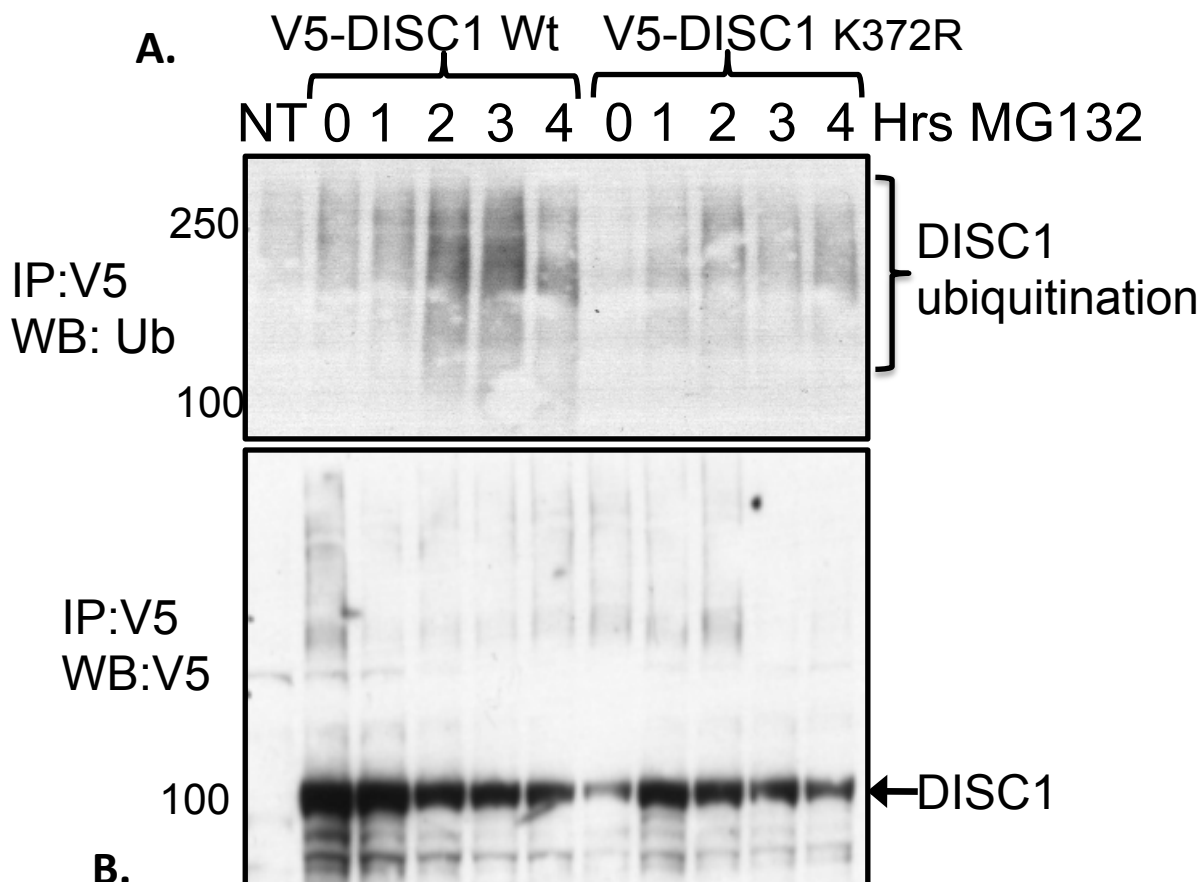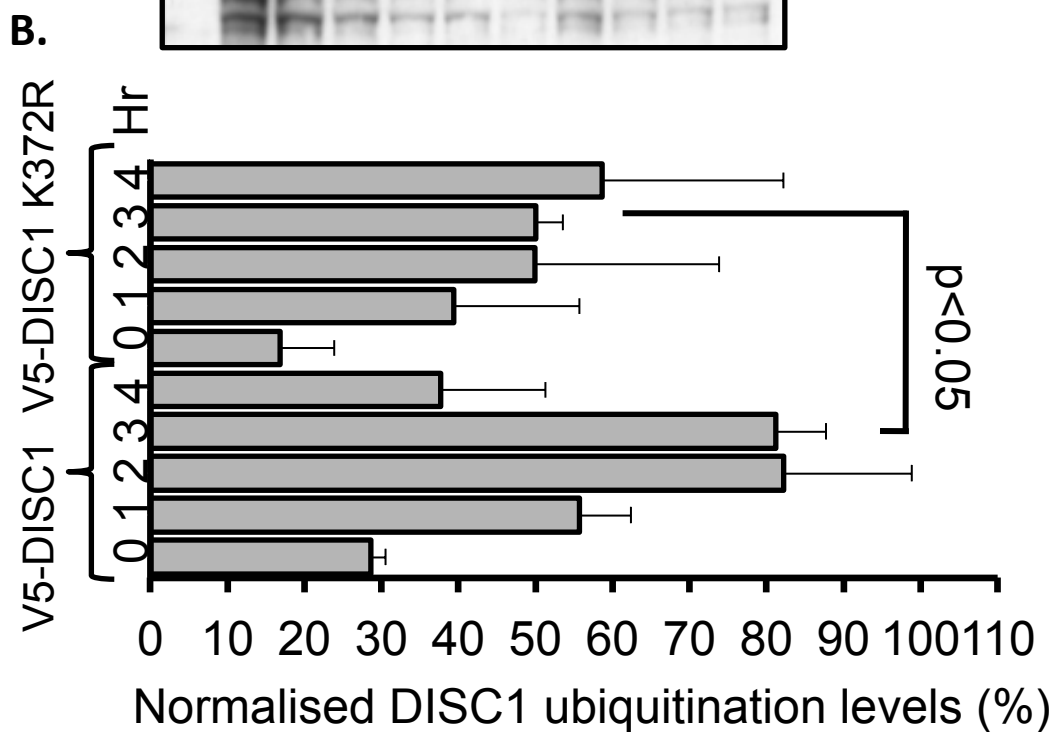

Supplement: Supplementary Figure 2 [file mp2017138x2.pdf]

**A.**

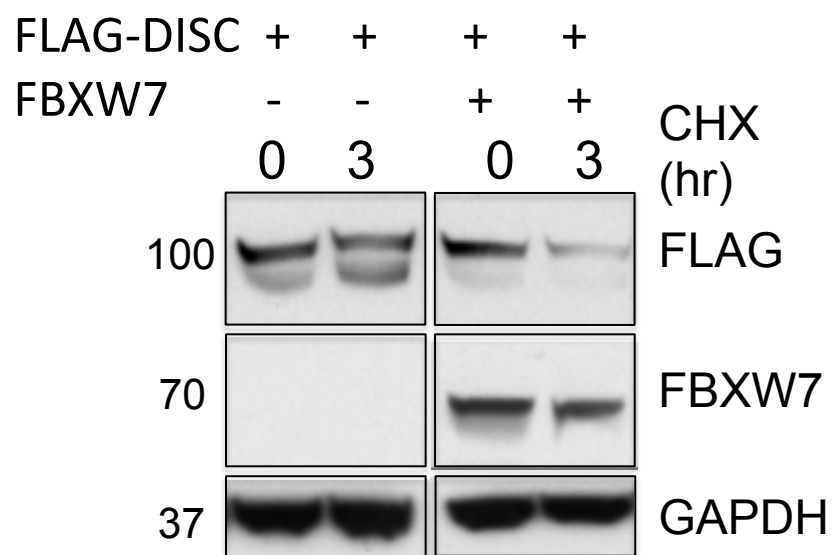

**B.** siR1 siR2 Con siCon siR3 siR4

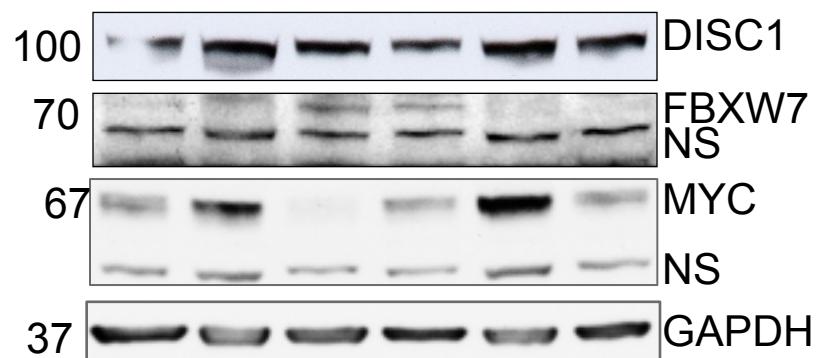

Supplement: Supplementary Figure 3 [file mp2017138x3.pdf]

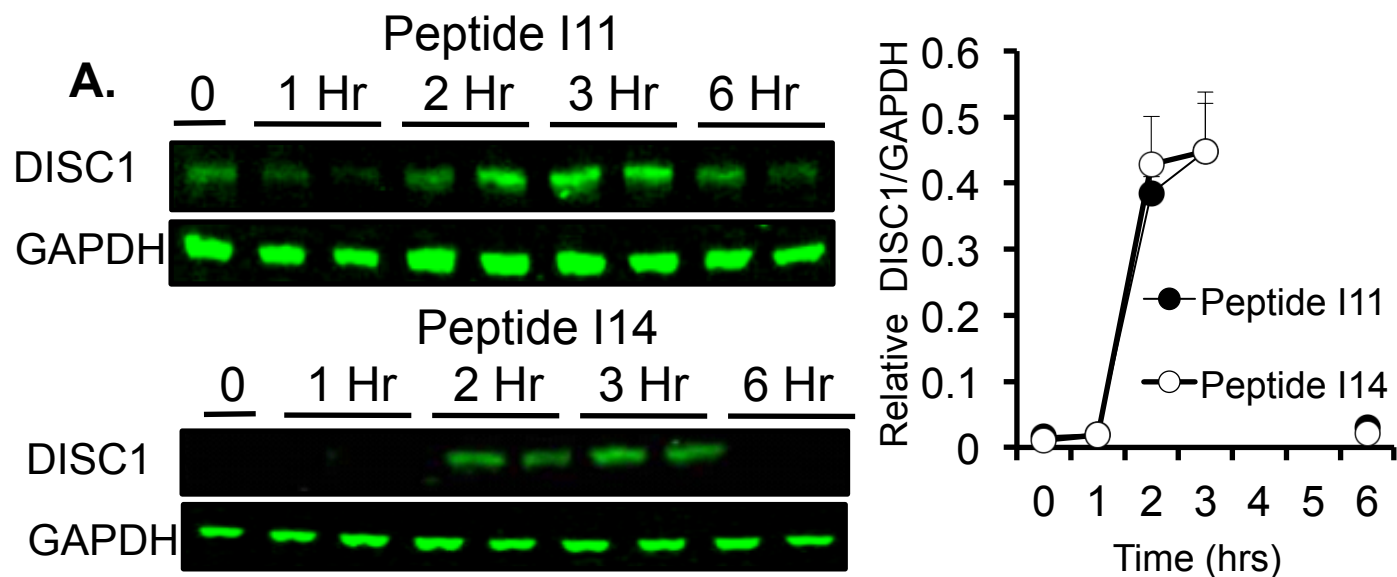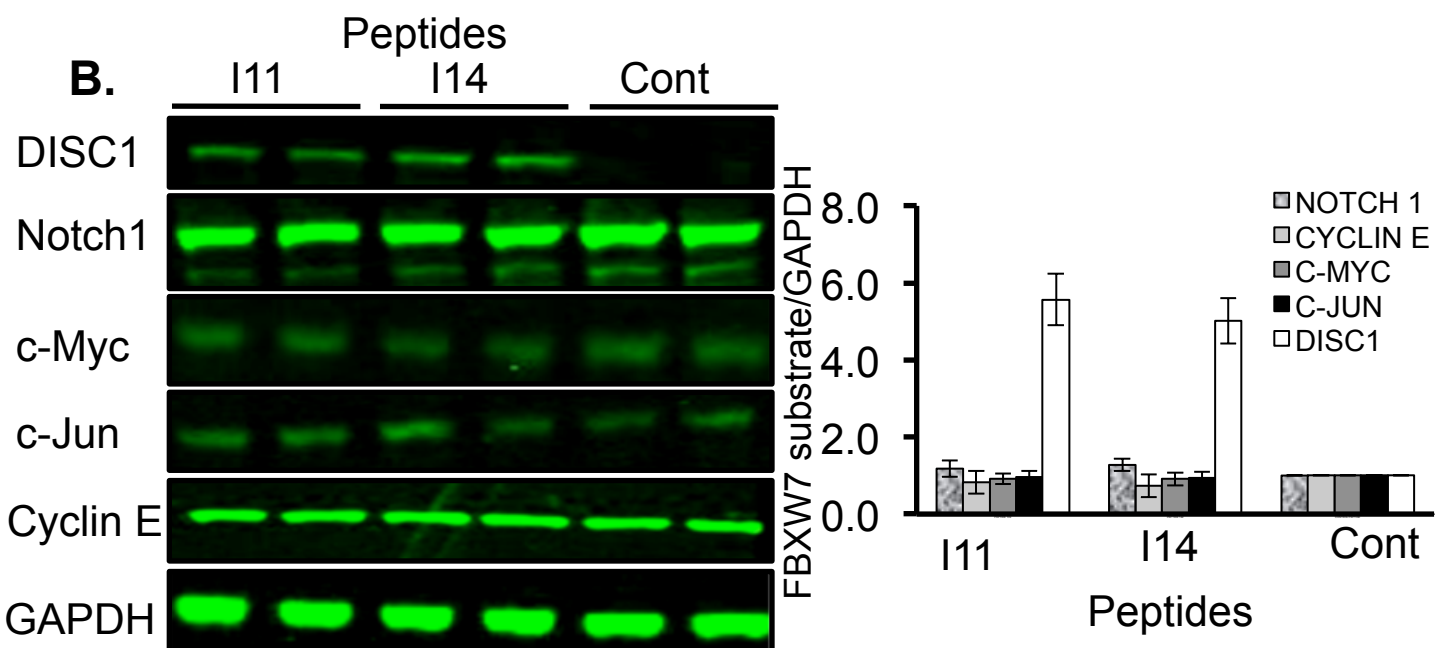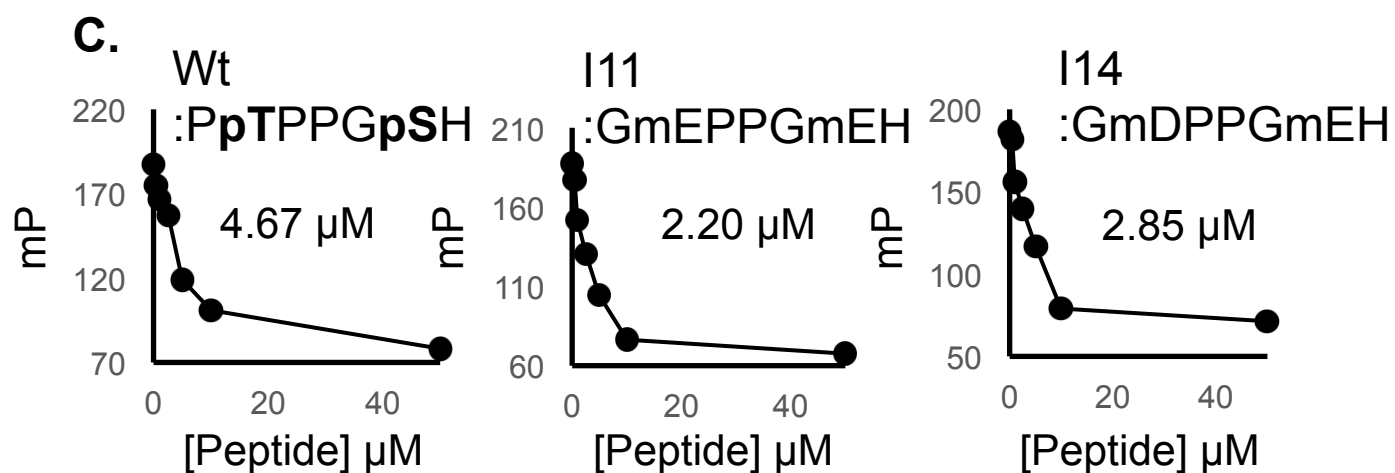

Supplement: Supplementary Figure 4 [file mp2017138x4.pdf]

**A**

**Fo-Fc map, 3.0  $\sigma$**

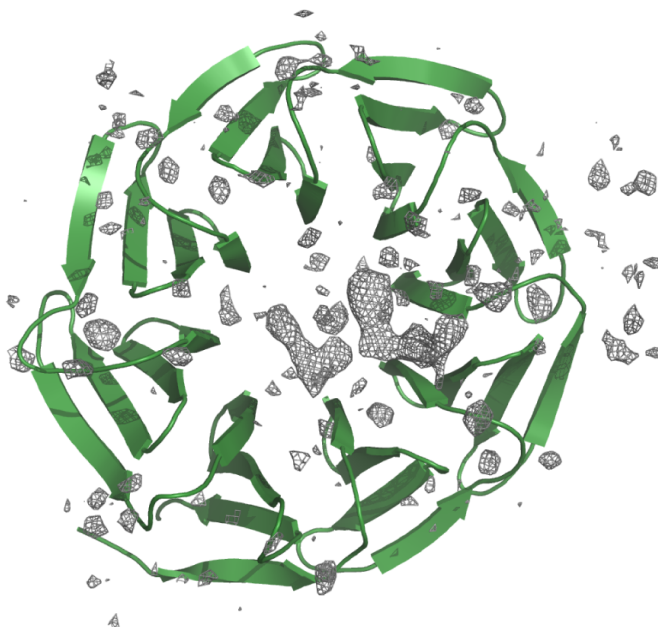

**B**

**2Fo-Fc map, 1.0  $\sigma$**

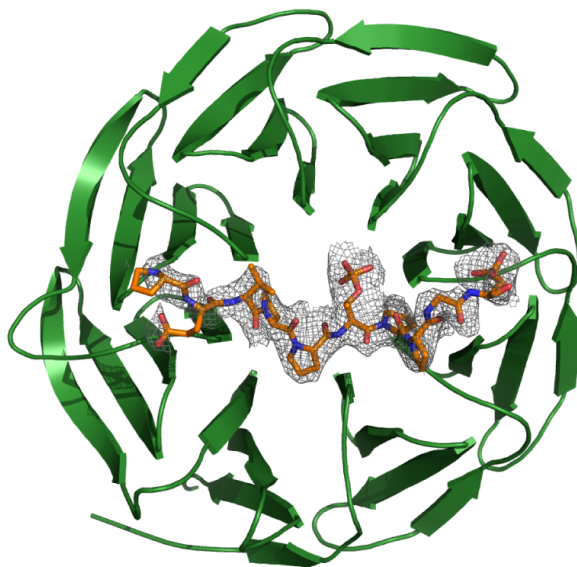

Supplement: Supplementary Figure 9 [file mp2017138x8.pdf]

## Slide 1
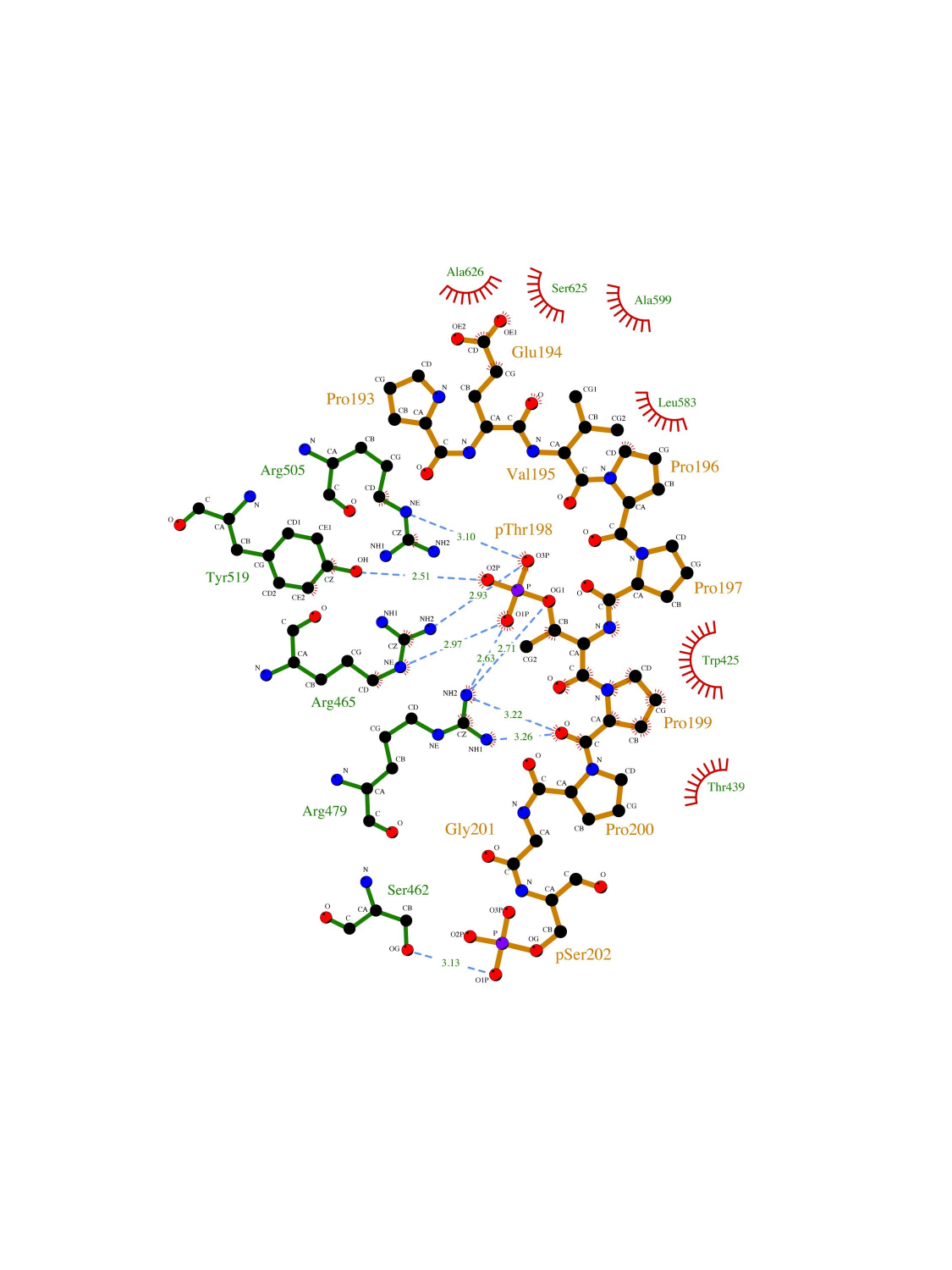

Supplement: Supplementary Figure 7 [file mp2017138x10.ppt]

## Slide 1
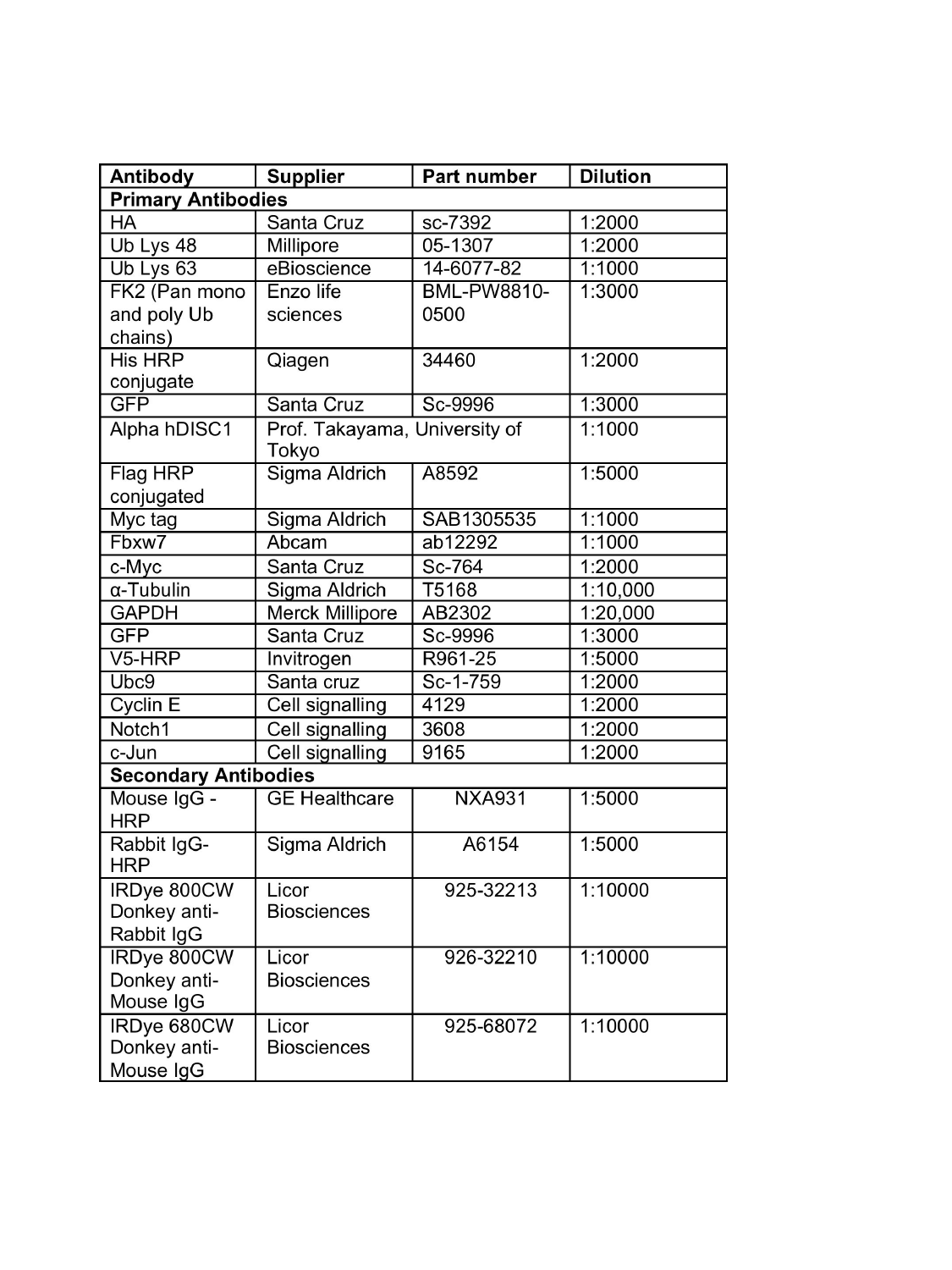

Supplement: Supplementary Table 1 [file mp2017138x11.ppt]

## Slide 1
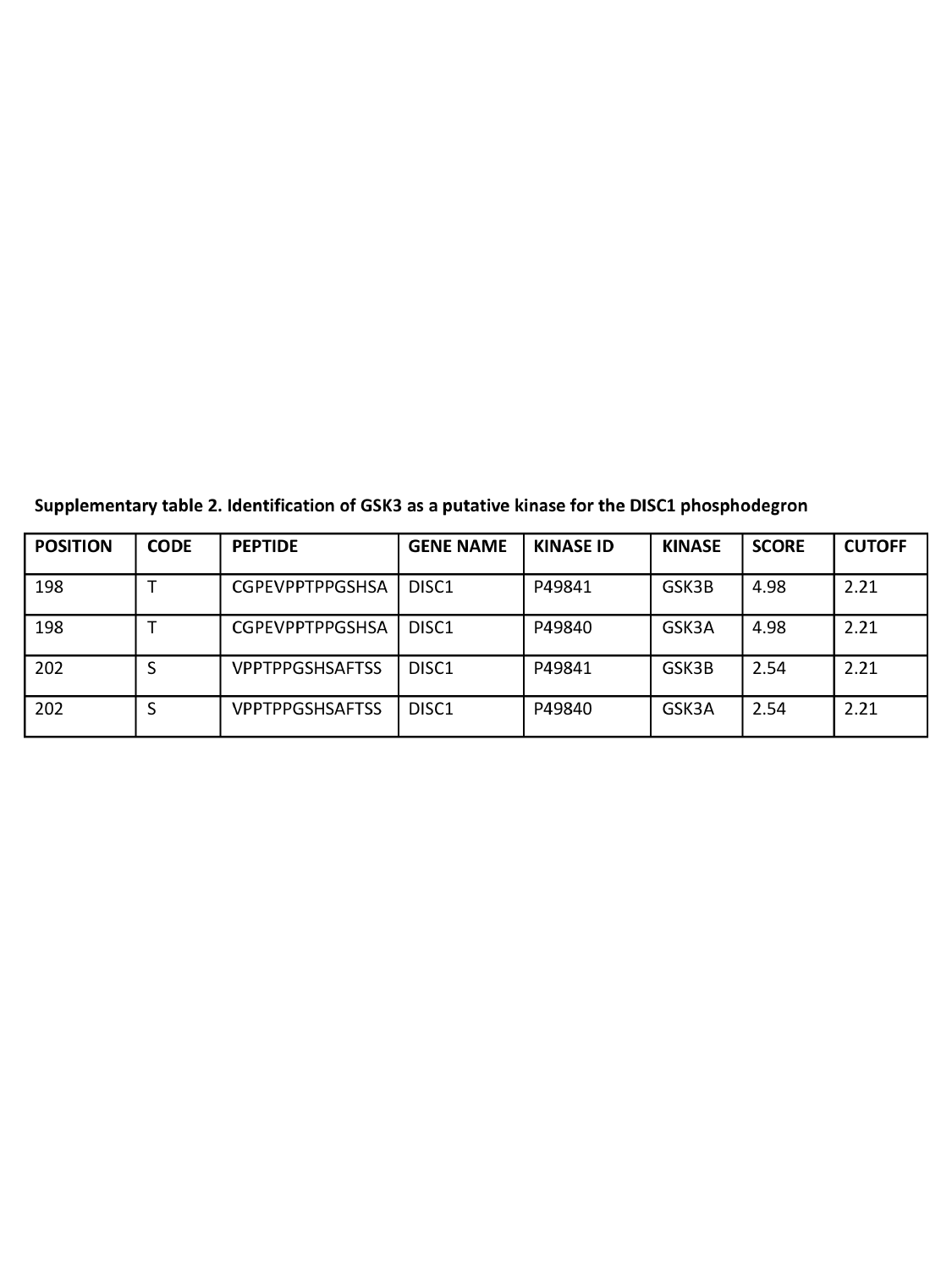

Supplement: Supplementary Table 2 [file mp2017138x12.ppt]
