# Supplementary material for: FBXW7 regulates DISC1 stability via the ubiquitin-proteosome system
Source: Mol Psychiatry. 2017 Jul 20;23(5):1278–86. doi: 10.1038/mp.2017.138 (PMC5984089; doi:10.1038/mp.2017.138)
Supplement: Supplementary Figure 5 [file mp2017138x5.pdf]

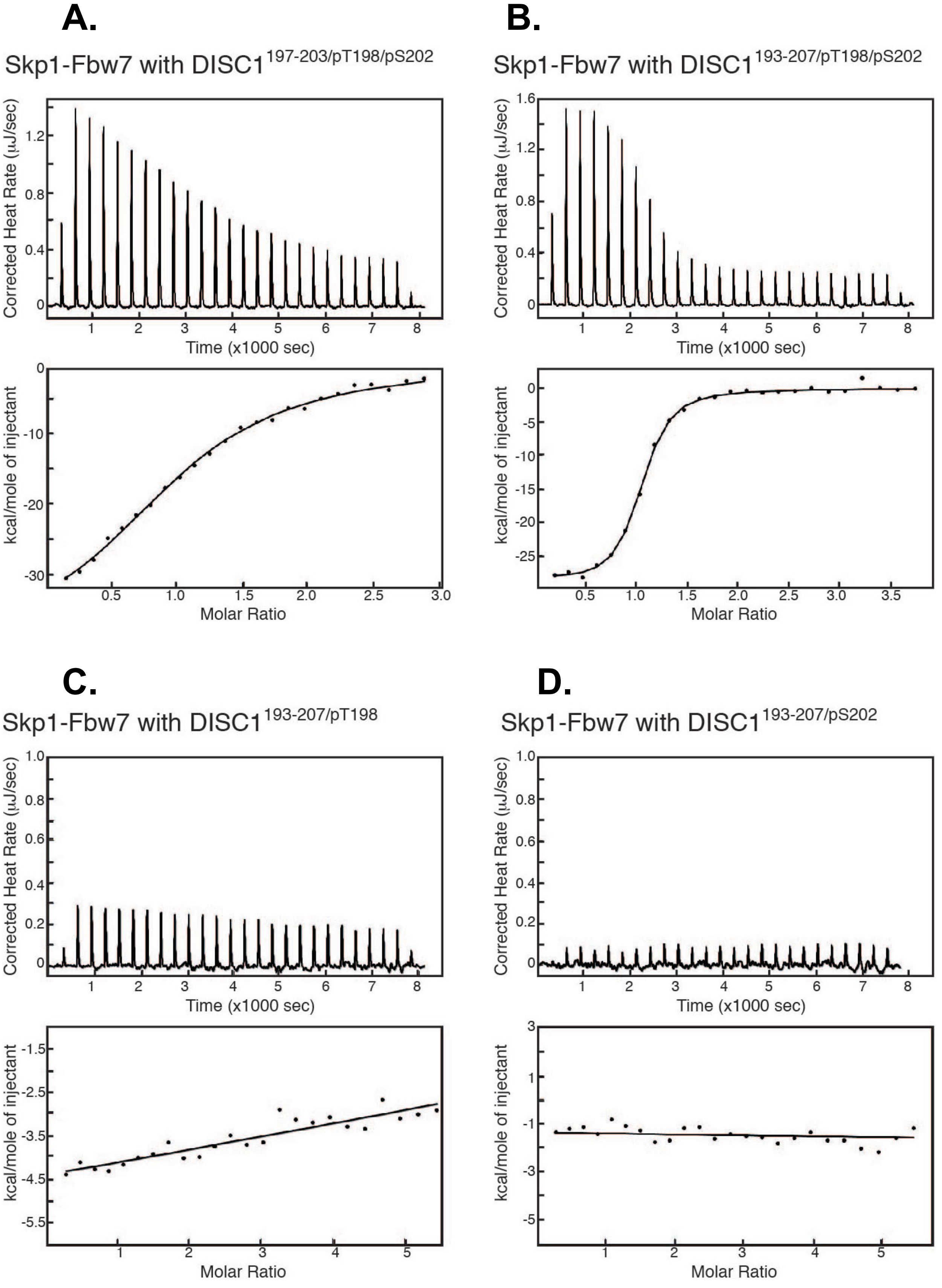
A.
Skp1-Fbw7 with DISC1$^{197-203/pT198/pS202}$
Corrected Heat Rate (μJ/sec)
Time (x1000 sec)
kcal/mole of injectant
Molar Ratio
B.
Skp1-Fbw7 with DISC1$^{193-207/pT198/pS202}$
Corrected Heat Rate (μJ/sec)
Time (x1000 sec)
kcal/mole of injectant
Molar Ratio
C.
Skp1-Fbw7 with DISC1$^{193-207/pT198}$
Corrected Heat Rate (μJ/sec)
Time (x1000 sec)
kcal/mole of injectant
Molar Ratio
D.
Skp1-Fbw7 with DISC1$^{193-207/pS202}$
Corrected Heat Rate (μJ/sec)
Time (x1000 sec)
kcal/mole of injectant
Molar Ratio
