# Supplementary material for: FBXW7 regulates DISC1 stability via the ubiquitin-proteosome system
Source: Mol Psychiatry. 2017 Jul 20;23(5):1278–86. doi: 10.1038/mp.2017.138 (PMC5984089; doi:10.1038/mp.2017.138)
Supplement: Supplementary Figure 6 [file mp2017138x6.pdf]

Data collection	Skp1-Fbw7-DISC1
Space group	$I4_122$
Cell dimensions (Å)	
a, *b*, *c* (Ǻ)	233.5, 233.5, 108.4
Resolution (Å)	50.0-2.6 (2.64-2.60)
R_{sym}	10.3 (60.5)
I/σ(I)	15.9 (2.7)
Completeness (%)	99.8 (99.4)
Redundancy	6.1 (5.7)
Refinement	
Resolution (Å)	42.9–2.6
No. of reflections	43,643
R_{work}/R_{free}	18.4/21.9
Total protein atoms	4,692
Water molecules	197
R.m.s deviations	
Bond lengths (Å)	0.015
Bond angles (°)	1.8
