# Supplementary material for: FBXW7 regulates DISC1 stability via the ubiquitin-proteosome system
Source: Mol Psychiatry. 2017 Jul 20;23(5):1278–86. doi: 10.1038/mp.2017.138 (PMC5984089; doi:10.1038/mp.2017.138)
Supplement: Supplementary Figure 8 [file mp2017138x7.pdf]

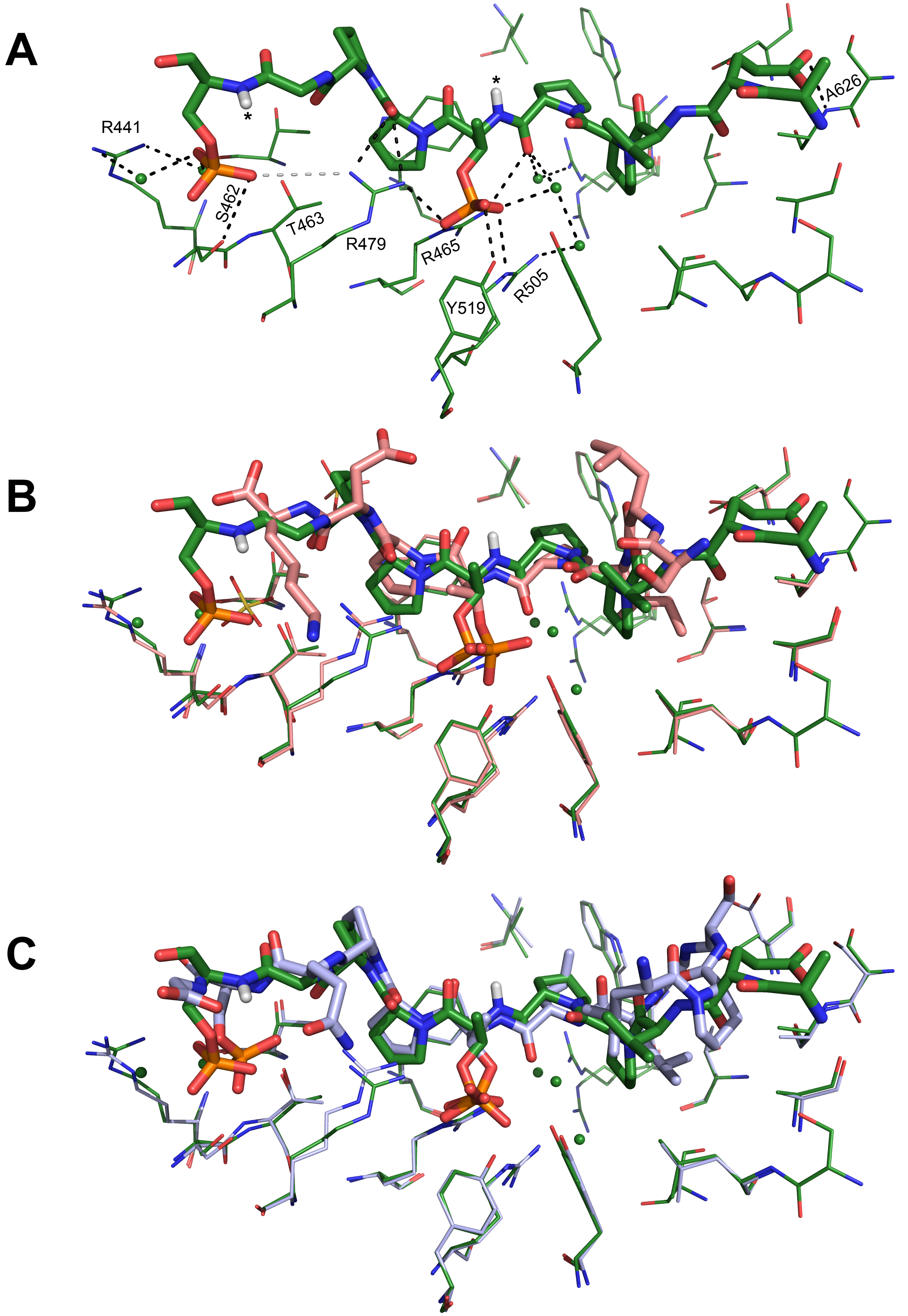

PHOSPHODEGRON SEQUENCES:

192	G	P	E	V	P	P	pT	P	P	G	pS	H	S	A	F	T	207 DISC1
071	P	C	S	L	I	P	pT	P	D	K	E	D	D	D	R	V	086 cyclin E N-ter
389	L	P	S	G	L	L	pT	P	P	Q	pS	G	K	K	Q	S	404 cyclin E C-ter
