# Supplementary material for: FBXW7 regulates DISC1 stability via the ubiquitin-proteosome system
Source: Mol Psychiatry. 2017 Jul 20;23(5):1278–86. doi: 10.1038/mp.2017.138 (PMC5984089; doi:10.1038/mp.2017.138)
Supplement: Supplementary Figure 10 [file mp2017138x9.pdf]

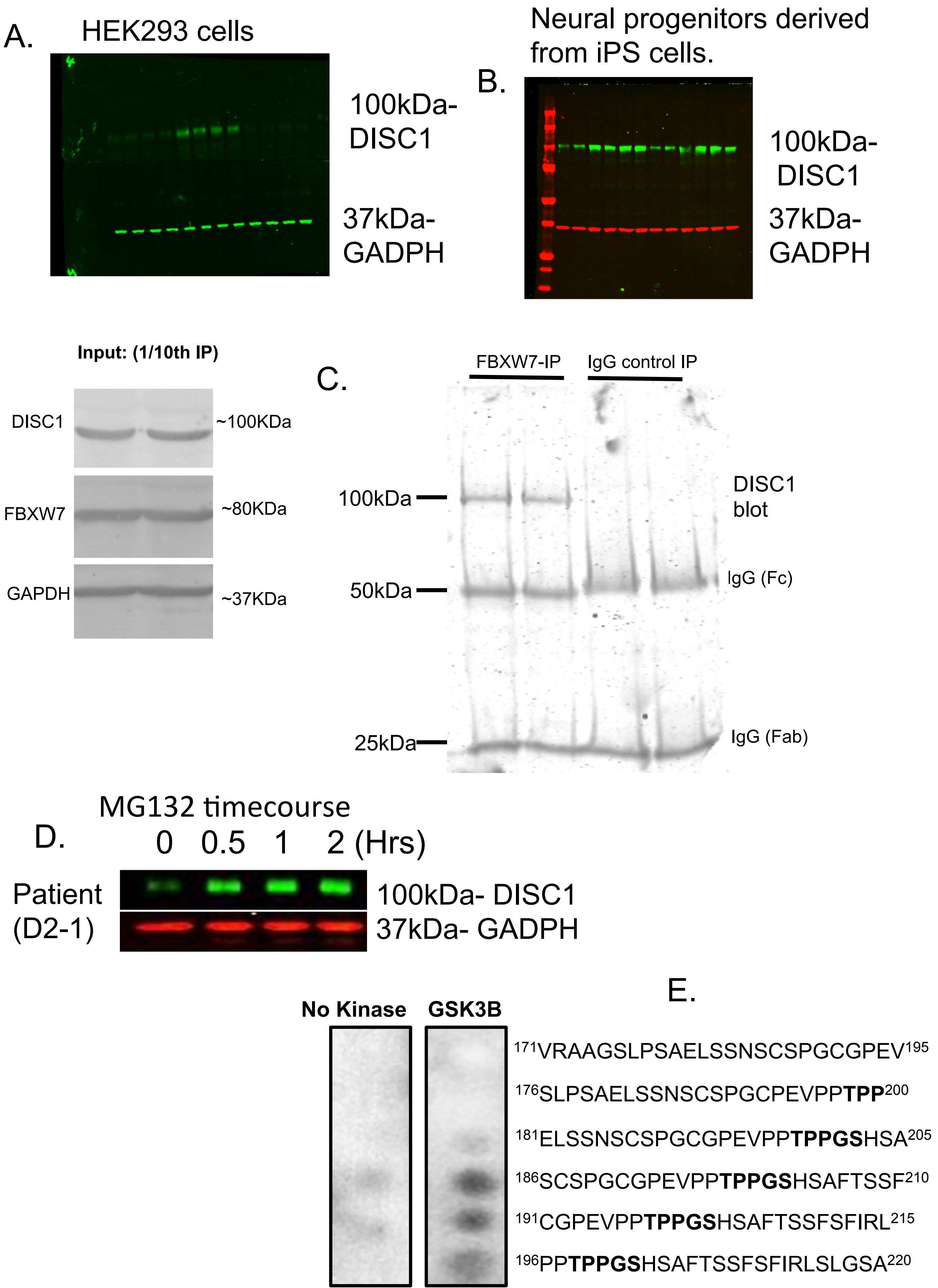
A.
HEK293 cells
100kDa-
DISC1
37kDa-
GADPH
B.
Neural progenitors derived from iPS cells.
100kDa-
DISC1
37kDa-
GADPH
Input: (1/10th IP)
DISC1
~100KDa
FBXW7
~80KDa
GAPDH
~37KDa
C.
FBXW7-IP
IgG control IP
100kDa
50kDa
25kDa
DISC1
blot
IgG (Fc)
IgG (Fab)
MG132 timecourse
D.
0
0.5
1
2 (Hrs)
Patient
(D2-1)
100kDa- DISC1
37kDa- GADPH
E.
No Kinase
GSK3B
171VRAAGSLPSAELSSNSCSPGCGPEV195
176SLPSAELSSNSCSPGCPEVPPTPP200
181ELSSNSCSPGCGPEVPPTPPGSHSA205
186SCSPGCGPEVPPTPPGSHSAFTSSF210
191CGPEVPPTPPGSHSAFTSSFSFIRL215
196PPTPPGSHSAFTSSFSFIRLSLGSA220
